# Sleeping through anything: The effects of unpredictable disruptions on mouse sleep, healing, and affect

**DOI:** 10.1371/journal.pone.0210620

**Published:** 2019-01-31

**Authors:** Amy Robinson-Junker, Bruce O’Hara, Abigail Durkes, Brianna Gaskill

**Affiliations:** 1 Department of Comparative Pathobiology, School of Veterinary Medicine, Purdue University, West Lafayette, Indiana, United States of America; 2 Department of Biology, University of Kentucky, Lexington, Kentucky, United States of America; 3 Department of Animal Sciences, Purdue University, West Lafayette, Indiana, United States of America; University of Portsmouth, UNITED KINGDOM

## Abstract

Many aspects of the laboratory environment are not tailored to the needs of rodents, which may cause stress. Unpredictable stressors can cause ulcers, prolonged pituitary-adrenal activation, and anhedonia. Similarly, pain has been demonstrated to slow wound healing, and mice experiencing pain exhibit altered behavior. However it is unknown how husbandry, which occurs when the mice are inactive, and lack of analgesia, specifically in a punch biopsy procedure, effects animal physiology, behavior, and welfare, particularly as it relates to sleep fragmentation. We hypothesized that sleep fragmentation, induced by unpredictable husbandry and lack of pain management will slow wound healing. Two main treatments were tested in a factorial design in C57BL/6 mice of both sexes (64 mice total); 1) analgesia (carprofen and saline) and 2) sleep disruptions (random and predictable). Mice were singly housed in a non-invasive sleep monitoring apparatus on arrival (Day -4). Disruption treatments were applied from Day -3 to 2. All mice received a punch biopsy surgery (Day 0) with topical lidocaine gel and their analgesic treatment prior to recovery, and on Days 1 and 2. Nesting behavior was assessed daily and a sugar cereal consumption test, as a measure of anhedonia, was conducted on Days -1 to 2. On Day 3, mice were euthanized and wound tissue and adrenal glands were collected. We found that the disruption predictability had no effect on mouse sleep, wound healing, or adrenal cortex:medulla ratio. It’s possible that the disruption period was not long enough to induce chronic stress. However, male mice who received analgesia slept more than their female counterparts; this may be related to sex differences in pain perception. Overall, it does not appear that the predictability of disturbance effects sleep fragmentation or stress responses, indicating that husbandry activities do not need to occur at set predictable times to improve welfare.

## Introduction

As reproducibility and successful translation of research findings become more difficult to achieve, the scientific community has begun looking for explanations and solutions. One particular area of interest has been the effect of the laboratory environment, and the animals’ experience of it, on research models [1–6]. The laboratory environment has been tailored to human preferences as much, if not more, than rodent needs. In turn, those unmet needs may then induce physiological outcomes that disrupt research activities. Mice experience cold stress at normal laboratory temperatures, depleting their energetic resources for reproduction[7–9]. Routinely provided corncob bedding is aversive and decreases sleep[10, 11], and typical handling[12, 13] induces stress and alters performance in behavioral tests. Furthermore, laboratory mice are nocturnal, but live in a diurnal environment to accommodate human workers. This raises a real risk that human activity during mouse rest periods is interfering with their sleep quality, quantity, or both. That interference may then, in turn, be altering research findings and complicating our ability to translate them to humans.

We have previously investigated the effect of timing (day or night) of husbandry disturbances (day vs night) [14]. We found that mice slept the same amount of time (both percentage of time spent sleeping and mean bout length) whether they were disturbed at 10:00 AM or 10:00 PM. However, the timing of their sleep shifted in response to disturbance timing, though in a very sex and strain or stock specific way. However, limiting entrance to the room to 1 time, in a specific 1 hour period of the day is impractical in a working vivarium setting. This led us to wonder how we could apply these findings in a usable manner. We thought that the unpredictability of human activity in the vivarium could affect mouse sleep in a way that a brief, predictable disruption did not. Of particular concern was sleep fragmentation (the interruption of sleep either through waking or transitioning to a lighter sleep stage). It can induce physiologic, metabolic, and (if experienced during gestation) epigenetic effects, including slowed wound healing[15–19]. However, there is a major gap in the literature regarding the impact of routine human activity on mouse sleep.

The ultimate function of sleep appears to be that of renewal. Anesthetized mice experience increased interstitial cerebrospinal fluid flow, which refreshes ADP into ATP and removes amyloid plaques [20]. Sleep is also a period of increased activity for pro-inflammatory cytokines, which assist the healing process [21–23]. Sleep even improves the immune response to vaccines [24]. So it should come as no surprise that sleep disruption could have serious impacts on mouse welfare and research outcomes.

What we know of sleep fragmentation in mice has typically come from studies using mice as a proxy for humans with sleep apnea or periodic limb movements [25]. Mechanized disruptions are generally used to induce frequent arousals from sleep (every 1–2 minutes) [15, 16, 26], rather than trying to mimic vivarium situations and experiences that mice are exposed to. In other words, we know about mouse sleep disruption when treating them like humans, but we don’t know much about it when treating them like mice.

Unpredictability is stressful for animals; rats who receive unpredictable shocks develop ulcers[27] and anhedonia[28], and rats given a choice will choose a predictable shock over an unpredictable shock[29, 30]. Typical vivariums involve multiple unpredictable disruptions. Animals from several projects may be housed in the same room, meaning researcher activities may not be coordinated. Running water, cleaning equipment, and even caretakers can vary on a daily basis. Not only are mice experiencing unpredictability of disruption, but these disruptions are also occurring during the light phase, when mice would ordinarily be sleeping. This combination may be sufficient to induce sleep fragmentation and stress.

One method of assessing the physiological effects of stress is through measuring wound healing; increased stress leads to slow or imperfect healing[31–38]. One stressor known to slow wound healing is pain [39–44]. Pain slows the healing process in humans [42–44] and alters general behavior; similarly, after experiencing a painful procedure, mice burrow less and build less complex nests[45–48], and are slower to incorporate new nesting material into an existing nest[49]. Additionally, pain interferes with sleep [50, 51], and sleep deprivation can induce hyperalgesia in rats[52]. This suggests that a vicious cycle may exist between these factors and requires that the interaction between pain, sleep, and healing be considered.

The effects of sleep disruption are not solely physiological. Work in both humans [53–56] and rodents [57] has shown cognitive changes after sleep deprivation and disruption, and sleep dysfunction is also associated with mood disorders in humans [58–60]. These findings indicate that an investigation of the potential welfare implications of sleep disruption should also include assessment of changes in mental well-being.

Our hypotheses were that unpredictable disruptions are more disruptive to mouse sleep than predictable disruptions. We also hypothesized that pain, following from lack of post-operative analgesia would negatively affect nesting behavior and sleep patterning. We predicted that mice who experienced frequent, unpredictable disruptions during their normal rest period would sleep less and/or have more fragmented sleep during the day and have stronger indicators of stress (decreased sleep, wound healing, and sucrose consumption) than those whose disruptions occurred at predictable times. We also predicted that mice who received analgesia (rather than a control injection) would sleep more during their normal rest period and have weaker indicators of stress.

## Materials and methods

### Ethical statement

This study was approved by the Purdue Animal Care and Use Committee (Protocol 1512001333), and conformed to all guidelines put forward by both the committee and the Guide for the Care and Use of Laboratory Animals [61]. At the start of study, animals were free of a list of common mouse infectious agents; further details may be found at http://www.criver.com/files/pdfs/rms/hmsummary.aspx. All mice were monitored daily by trained members of the research team for food and water consumption and overall health status, with no adverse conditions or health outcomes noted.

### Experimental design, animals, and housing

Two main treatments, in a factorial design, were assessed in naive C57BL/6NCrl mice of both sexes (6 weeks of age; Charles River, Kingston, NY); 1) sleep disruption (unpredictable or predictable) and 2) analgesia administration (analgesia and saline). Each factorial combination had four replicates for a total of 32 mice (Table 1). Mice were tested from May to June of 2016.

**Table 1:** Experimental factorial design.

Disruptions	Predictable				Unpredictable			
Sex	Male		Female		Male		Female	
Analgesia	Y	N	Y	N	Y	N	Y	N
Replicates	4	4	4	4	4	4	4	4

Factorial design with number of replicates (mice) per combination of treatments. Mead’s rule was used to determine the number of mice needed based on our experimental design.

**Fig 1:**
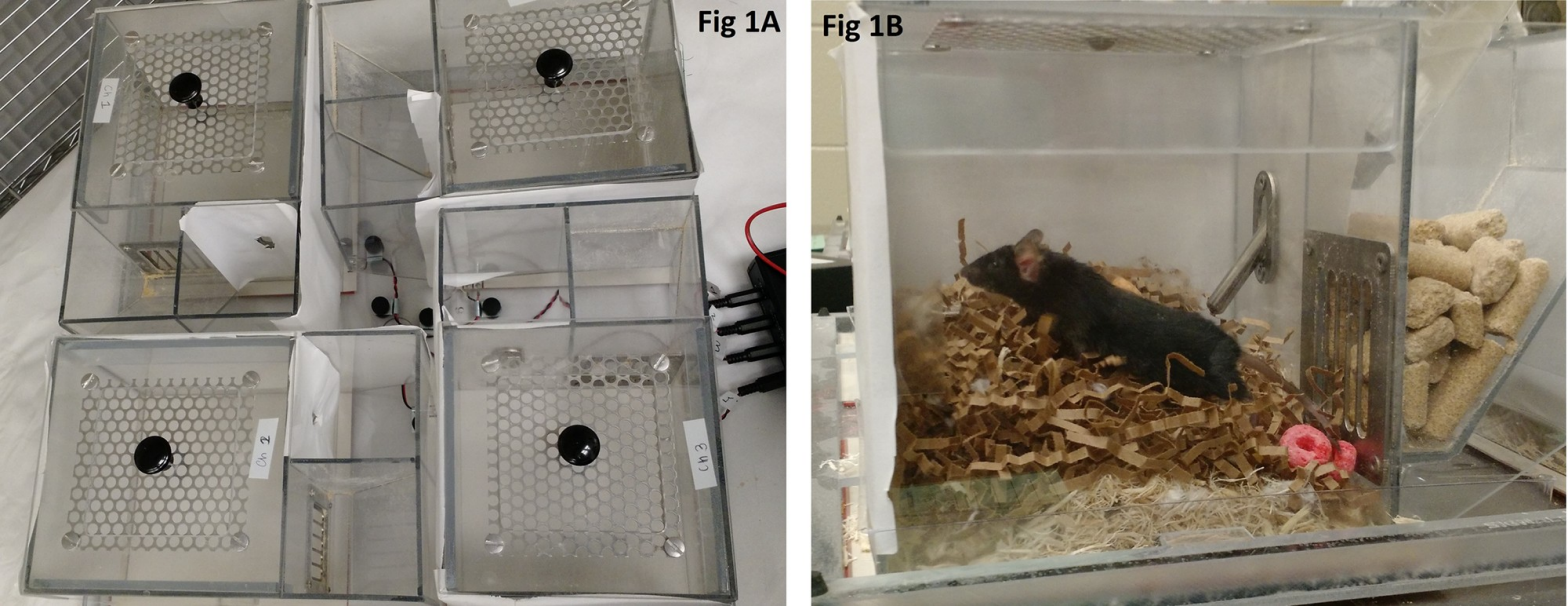
Sleep apparatus viewed from above (A) and a close up side view of an individual mouse cage (B). Sugary cereal used for the sucrose preference test can be seen in B.

Mice were housed in one of the two sleep monitoring apparatuses (Fig 1). Each apparatus houses 4 mice, each in a separate chamber; this allowed us to test 8 mice simultaneously. The apparatus uses a piezoelectric mat underneath each cage to detect vibrational movement of the mouse and therefore mice must be housed singly. Customized software (MouseRec Data Toolbox, Signal Solutions, Lexington KY) uses an algorithm to process the signal and discern sleeping respiratory patterns from waking respiratory patterns; this algorithm has been validated using EEG, EMG, and visual evaluation [62]. A different algorithm also permits quantification of activity level, where higher numbers indicate greater intensity of activity; we used this measure in an effort to discern whether awake mice changed their activity levels.

**Fig 2:**
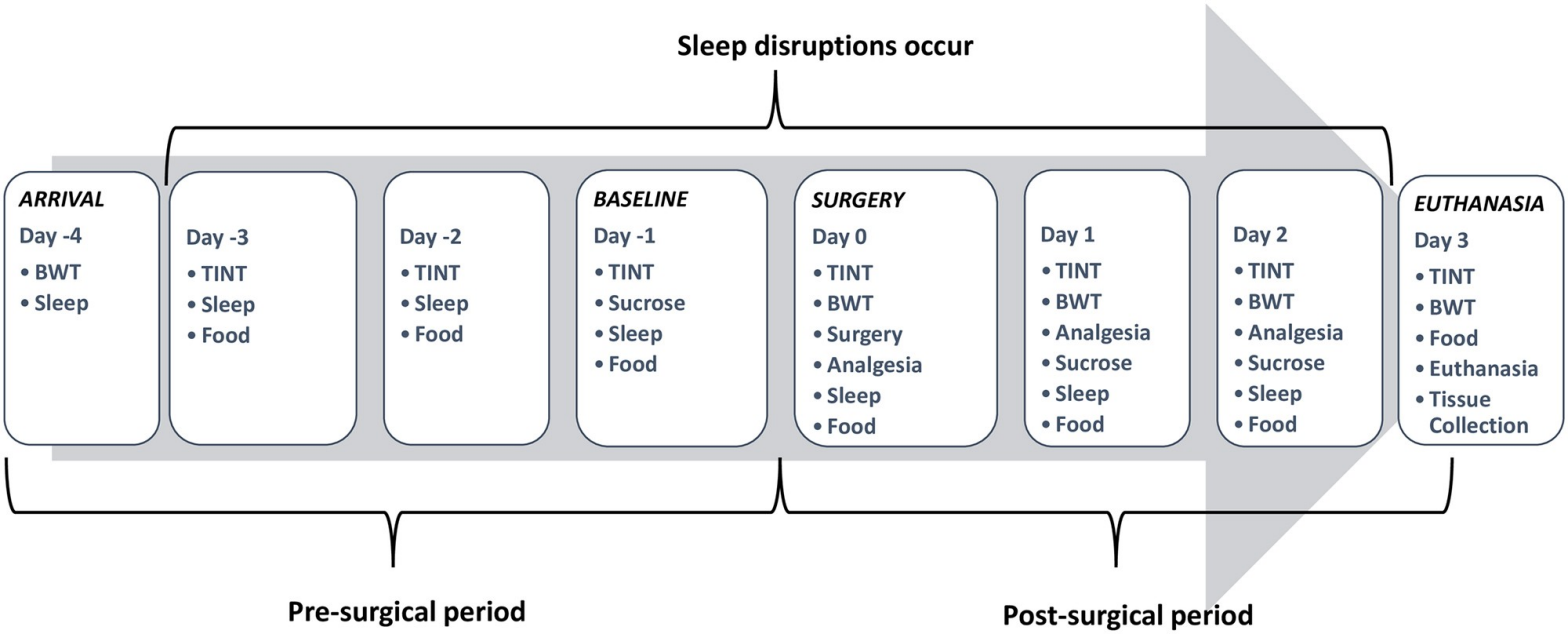
Experimental timeline. Lists all measurements made on each day of experiment. Day -1 is considered baseline. Mice arrive on Day -4. Abbreviations: **BWT**—bodyweight; **Sleep**—sleep monitoring; **TINT**—Time to Integrate Nesting Material Test; **Food**—food consumption; **Sucrose**—sucrose consumption; **Analgesia**—analgesia treatment.

Visual barriers were in place between cages, but audible and olfactory contact was still possible. Each cage included a built in food hopper and water bottle opening. Each cage (6.0 in x 6.1 in x 6.1 in) was bedded with 32g of laboratory grade aspen shavings (Harlan, Indianapolis IN) and 8g of nesting material (Enviro Dri, Shepherd Specialty Papers, Watertown, TN). Mice were provided with an 18% protein laboratory diet (Harlan 2018, Indianapolis IN) and reverse osmosis filtered water *ad libitum*. Lights were kept on a 12:12 light/dark cycle, with lights on at 05:00 and off at 17:00 hours. The room was maintained at 72± 2 F, and 36–64% humidity. Upon arrival (Day -4 –see Fig 2), mice were randomly assigned to an analgesia treatment using a random number generator (www.random.org). The experimenter was not blinded as to their assignment, because the experimenter also prepared and administered the medications. Mice were weighed and placed in their cage within the sleep apparatus no longer than 1 hour before lights out.

#### Disruption treatments

Sleep disruptions began immediately after arrival. Because all testing was conducted in a single room, all 8 mice in a test group were exposed to the same disruption treatment (unpredictable or predictable) simultaneously. Both treatments consisted of the same 8 disruptions—presence of a stranger, a recorded conversation playing in the room, a radio playing pop music, cage changing noises, presence of a t-shirt that was worn by a man, running water, a running cage changing station with ventilation hood, and floor disinfection (Table 2). These disruptions were chosen based upon activities that occur in a typical vivarium and factors that are known to alter mouse behavior (such as the presence of a male investigator, or a shirt worn by one [63]). The order and duration of the disruptions were initially scheduled in a random fashion, but the schedule itself was consistent across disruption replicates. For instance, all mice experienced the same disruptions on the same day during the experiment, for the same durations. The only difference was whether they were spread randomly throughout the day (unpredictable) or consolidated at the beginning and end of the day (predictable). No disruption was repeated in the same day, and there were a total of 4 disruptions per day. Potential durations of disruption were 15, 30, 45, or 60 minutes; floor disinfection and running water only lasted 15 minutes due to practical and environmental considerations. In the unpredictable disruption group, the interval between disruptions was also randomized, with intervals between them of either 45, 60, 90, or 120 minutes. For the predictable disruption group, disruptions occurred between 2.5–3.5 hours after lights on (7:30–8:30) or within an hour of lights off (16:00–17:00), with two disruptions scheduled for the morning period, and two for the evening period. The exception to this schedule was on the morning of the punch biopsy procedure; no disruptions were scheduled that morning.

**Table 2:** Sleep disruptions and durations.

Disruption	Description	Duration	Number of Occurrences
Cage change	Investigator removes mice from cage, supplies fresh bedding and nesting material, replaces mouse	30 min	1
Cage change noise	Investigator rattles cages containing corncob bedding and lids	45 min, 60 min	3 (45 min x1, 60 min x2)
Conversation	Smartphone used to play back each of two specific stand up comedy tracks (65–72 dB at cage level)	45 min	2
Exhaust fan	Exhaust fan of cage changing station turned on (62 dB at cage level)	30 min, 60 min	3 (30 min x1, 60 min x2)
Floor cleaning	Investigator uses power washer to distribute cleaning solution, scrubs floors with scrub brush, rinses with bucketed water, then squeegees floor dry	15 min	1
Male t-shirt	Investigator places t-shirt that was worn the night before in the room near the cages	30 min, 60 min	3 (30 min x1, 60 min x2)
Music	Antenna radio tuned to local rock music station	15 min, 30 min, 45 min, 60 min	4
Running water	Water left running in stainless steel sink (58–62 dB at cage level)	15 min	3
Stranger	Unfamiliar person sits or stands quietly in room without interacting with mice	15 min, 45 min	2
Unfamiliar smell	Investigator sits quietly in room while wearing strongly scented lotion	30 min	1

Disruption descriptions, durations, and number of occurrences. When disruptions occurred more than once, different durations were possible; if that was the case, all duration times are listed. All mice experienced all disruptions.

#### Analgesia treatment

Mice assigned to the analgesia treatment group received 10mg/kg carprofen subcutaneously on Day 0 (after wounding), Day 1, and Day 2. Mice in the analgesia control group received an equal volume of saline subcutaneously on the same days as the analgesia mice. On Day 1 and 2, a dorsal access mouse restrainer (Braintree Scientific, Braintree MA) was used to hold the mice while an investigator administered a subcutaneous injections in the caudal region, avoiding manipulation of the surgical area and the potential risk of medication leaking from the surgical site.

#### Punch biopsy procedure

After 4 days of disruptions (Day 0), all mice were anesthetized with isoflurane in an induction chamber and maintained on isoflurane administered via nose cone. We clipped and sterilized the cervical area of each mouse, placed them in lateral recumbency, and pulled the dorsal skin away from the animal, as if scruffing them. We then utilized a 3mm biopsy punch (Sklar Surgical Instruments, West Chester, PA) to push through both layers of skin, creating 2 symmetrical 3mm full-thickness wounds. The wounds were not sutured, stapled, or glued. Surgical order was balanced to account for order effects. During this procedure, we used a chemical hand-warmer (HotHands, Kobayashi Americas, Dalton GA) to provide thermal support to the mice. All mice, regardless of analgesia group, received 0.05 mL of 2% lidocaine gel topically applied to each wound for short-term local analgesia. Mice then received their assigned analgesic treatment. Mice were then moved to heated recovery cages until they were ambulating normally. Once recovered, they were returned to their home cage in the sleep apparatus. Two post-operative health checks were performed two hours apart. Sleep disruptions resumed as scheduled that afternoon.

#### Behavioral testing

Sleep and activity data were collected continuously via the sleep apparatus. We began data collection once the final mouse was housed on Day -4 (prior to 17:00) and ended by 9:00 the morning of Day 3, prior to euthanasia.

Mice were TINT tested[49] to assess pain and general welfare. In brief, in the TINT we provide a small amount of new nesting material to mice 2–3 hours after lights on and gave them 10 minutes to integrate this new material into their existing nest. A positive TINT score means the material has been incorporated, and suggests positive welfare. A negative TINT score suggests that the mice in that cage may be experiencing poor welfare, and personnel should investigate further. For this project, an investigator would enter the room, cut a Nestlet (Ancare, Bellmore, NY) into 4 equal squares, deliver one piece to each cage, and leave the room for 10 minutes. Upon returning to the room, the investigator assessed whether or not the material had been integrated into the nest. In this case, ‘integrated’ means ‘had been transported to the main body of the nest’. TINT testing occurred daily at 8:00 AM. This time corresponds with peak nest-building behavior [48]. The scores on Days -3 to -1 were considered ‘practice’, as mice have been shown to shorten their latency to incorporate material with repeated exposures [49, 64], so data presented from Day -1 is used as their baseline TINT.

Sucrose preference testing was used to assess anhedonia[65]. We did this by providing mice with 5g of sugary cereal (Froot Loops, Kelloggs, Battle Creek MI; a fruit-flavored breakfast cereal with approximately 12 g of sugar per 29 g of cereal) between 16:00 and 17:00 (prior to lights out), and then weighing the remainder between 7:30 and 8:00 the next morning. This allowed us to calculate the amount of cereal consumed each night; a decrease in consumption is indicative of anhedonia. We conducted these tests on Day -1, Day 1, and Day 2.

#### Sample collection

On Day 3, mice were euthanized via carbon dioxide. Immediately after euthanasia, mice were weighed and the punch biopsy area was excised, as well as surrounding tissues. Adrenal glands were also collected, in order to assess HPA axis activation[66, 67]. All tissues were fixed in 10% neutral buffered formalin and embedded in paraffin. Sections 5 μm thick were stained with haematoxylin and eosin according to standard methods. Microscopic examination was performed by a board-certified veterinary pathologist and the interpretation was based on standard histopathological morphology. The pathologist (AD) was blinded to the treatment groups. Wound width and re-epithelialization were quantified for all mice. Wound width was defined as the distance between wound margins in which the original epidermis was intact. Re-epithelialization was calculated as amount of newly formed epidermis as a percentage of the wound margin. Newly formed epidermis was defined as less than 3 cell layers thick of squamous epithelium devoid of stratum corneum.

Adrenal glands were sectioned en toto and representative sections were cut 50 micrometers deep. One adrenal gland per mouse was used to calculate an average cortex to medulla length ratio. Three cortical lengths and 3 cross-sectional medulla lengths were averaged and a ratio was calculated for each mouse.

### Data analysis

The experimental unit in all analyses was the individual mouse, and main treatments were disruption and analgesia. All data, with the exception of TINT success/failure, were analyzed using up to 3^rd^ degree factorial General Linear Model (GLM) in JMP (version 11, SAS Institute Inc) of the following factors: sex, disruption treatment, analgesia treatment, experiment day and (for sleep and activity data) lights on or off. To calculate food consumption, regular diet and sucrose cereal consumption were combined to calculate the total intake, where applicable. Individual mouse was the experimental unit and was used as a random factor, with sex, disruption treatment, and analgesia treatment nested within it. Cage location and sleep apparatus were used as blocking factors. Bodyweight was included as a covariate with food consumption, sucrose consumption, and adrenal cortex:medulla ratio. We used square root transformation for sleep bout length and activity level data, and log transformation for adrenal cortex:medulla ratio, in order to meet the assumptions of GLM. The assumptions of GLM (normality of error, homogeneity of variance, and linearity) were confirmed post-hoc[68]. Significant effects were then analyzed using post-hoc Tukey tests. All values are given as least squares means ± standard error (LSM ± SE).

TINT success/failure was analyzed using up to 3^rd^ degree factorial Generalized Linear Model (GLIM) for binomial logistic regression, with Firth-adjusted bias, for the following factors: sex, disruption treatment, analgesia treatment, and day of experiment. Cage was used as a fixed factor, with sex, disruption treatment, and analgesia treatment nested within it. Cage location and apparatus number were used as blocking factors. Non-significant 3^rd^ degree interactions were removed from the model, which produced a lower AICc number, denoting an improved model fit[69]. The full model had an AICc number of 220.972, while the reduced model’s number was 215.399. Pairwise planned contrasts were subsequently conducted on levels of significant factors to assess where differences arose, and were Bonferroni corrected for multiple comparisons.

For sleep measures, we excluded data from Day 0 because mice underwent surgery that day and that would alter sleep in a manner unrelated to our main treatments.

## Results

### Sleep measures

#### Proportion of time spent sleeping

**Fig 3:**
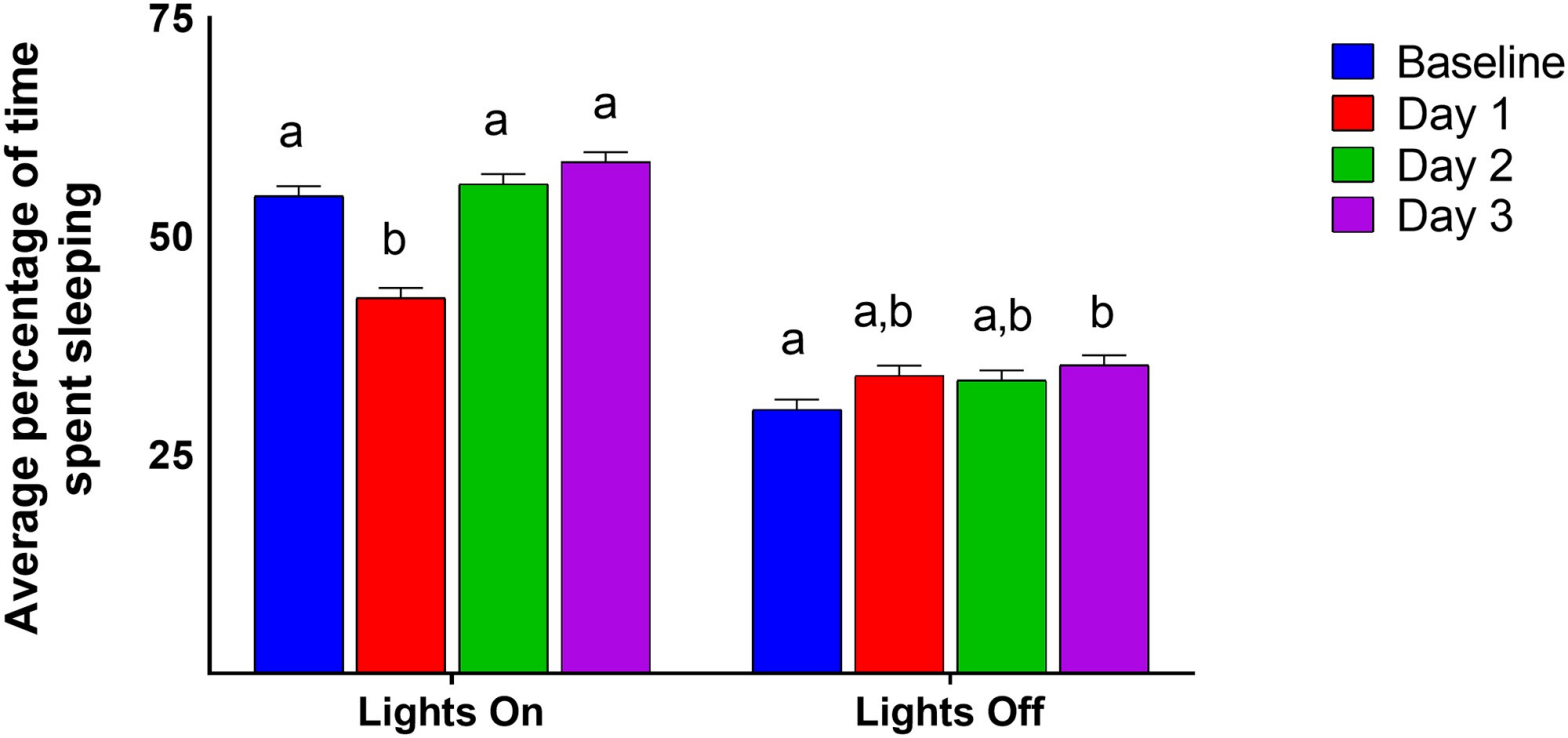
Average percentage of time spent sleeping by light phase and day of experiment. Different letters indicate significant (Tukey, P < 0.05) differences within categories. Data presented are LSM and SE.

We found multiple effects on proportion of time spent sleeping. A sex by analgesia treatment interaction (GLM, F_(1, 21)_ = 6.38, P = 0.0196) indicated that males who received analgesia slept more than females with analgesia. However, no other differences between control animals or within the sex were observed. An interaction of sex and whether the lights were on or off was also significant (GLM, F_(1, 184)_ = 5.34, P = 0.0219), with males sleeping more during lights off than females. Further a light phase by day in experiment interaction (GLM, F_(3, 184)_ = 26.99, P < 0.0001; Fig 3) showed that animals slept less when the lights were on during Day 1 than Day -1, 2, or 3. Additionally, animals slept less during lights off during Day -1 than they did on Days 2 and 3. And mice slept more during lights on than lights off during Days 1, 2, and 3. Finally, a 3 way interaction between disturbance treatment, analgesia treatment, and light phase was significant (GLM, F_(1, 184)_ = 14.32, P = 0.0002). However, this effect was solely due to light phase, with mice sleeping more when lights were on.

#### Sleep bout length

**Fig 4:**
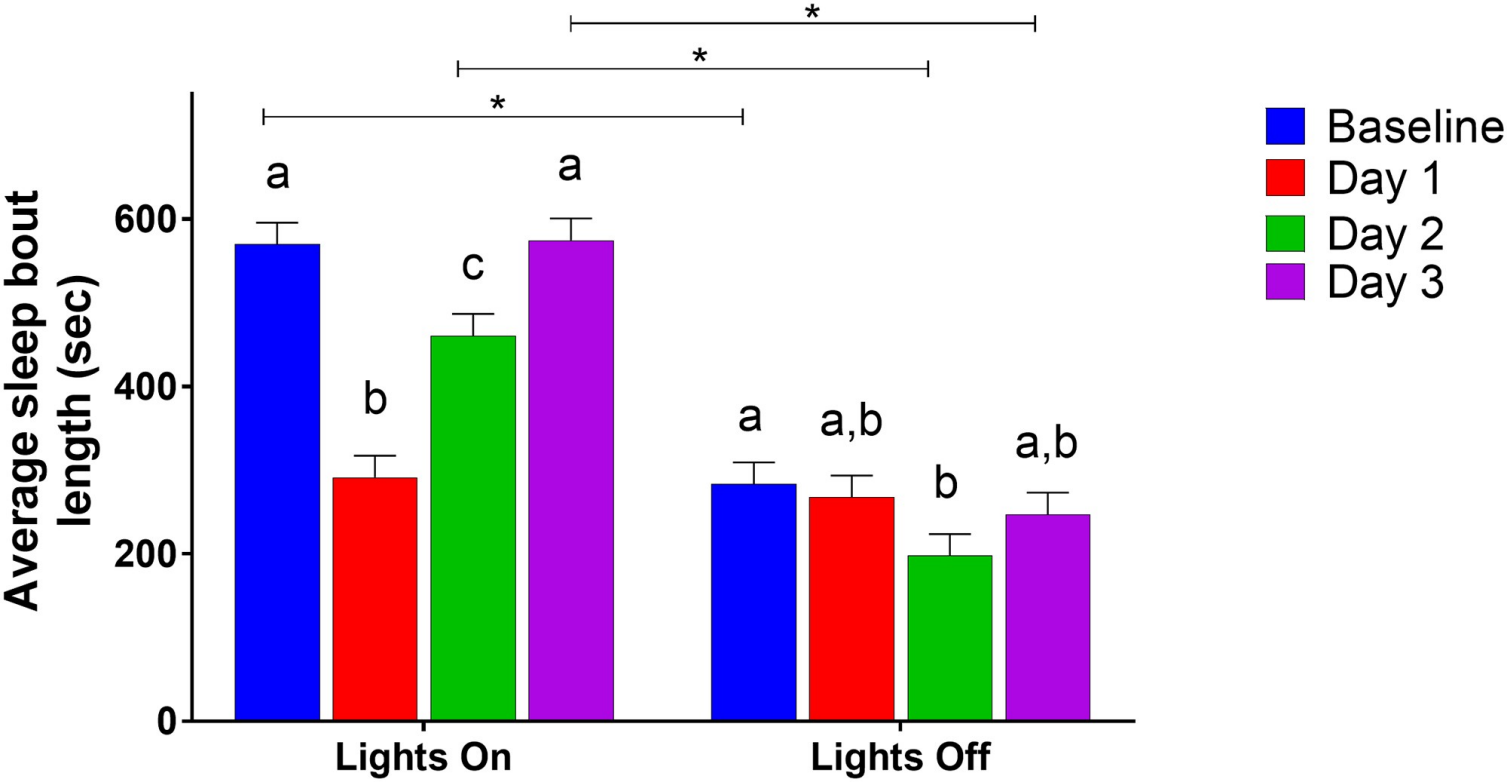
Average sleep bout length by lights on/off and day of experiment. Different letters indicate significant (Tukey, P < 0.05) differences within categories, bars indicate differences between categories. Data presented are LSM and SE.

Mean sleep bout length had multiple significant interactions. Light phase by the day of experiment (GLM, F_(3, 184)_ = 18.42, P < 0.0001; Fig 4) showed that, during lights on, mice had the shortest bout lengths on Day 1; during lights out, their bout lengths were shortest on Day 2. There was also a significant interaction between sex, analgesia treatment, and lights on/off (GLM, F_(1, 184)_ = 4.48, P = 0.0356). However, post-hoc Tukey analysis showed no differences between groups. Sex by analgesia treatment (GLM, F_(1,78.49)_ = 5.59, P = 0.0205) showed that female mice who received analgesia had shorter sleep bouts than those in the control group; there was no difference in the male mice, or within treatments.

### Activity levels

**Fig 5:**
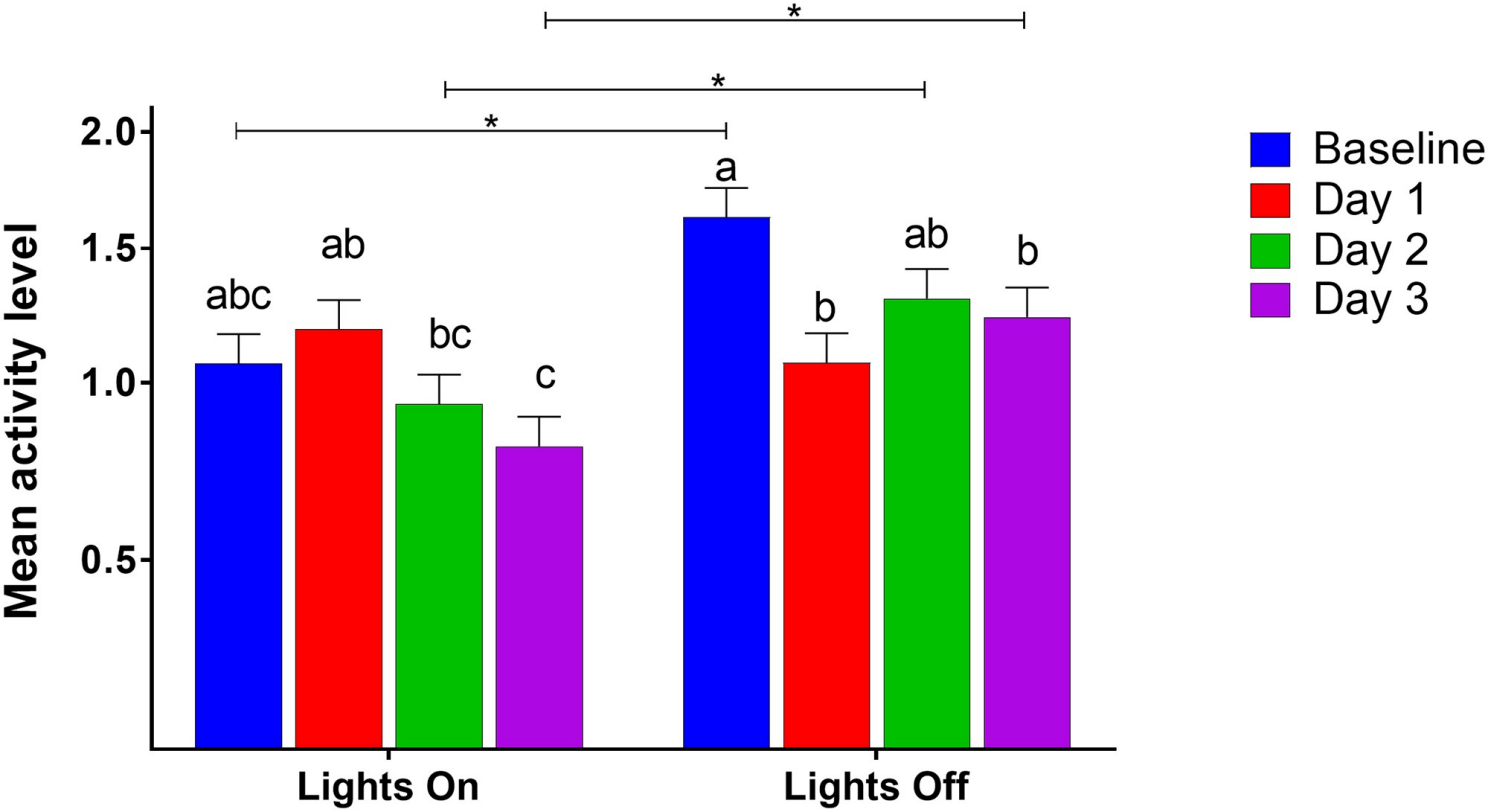
Mean activity level by light phase and day of experiment. Different letters indicate significant differences within categories, bars with asterisks indicate differences between categories (Tukey, P < 0.05). Data were square root transformed for analysis; y-axis is back-transformed. Activity level is a linear measurement from 0 to 3; higher values indicate higher levels of activity. Data presented are LSM and SE.

**Fig 6:**
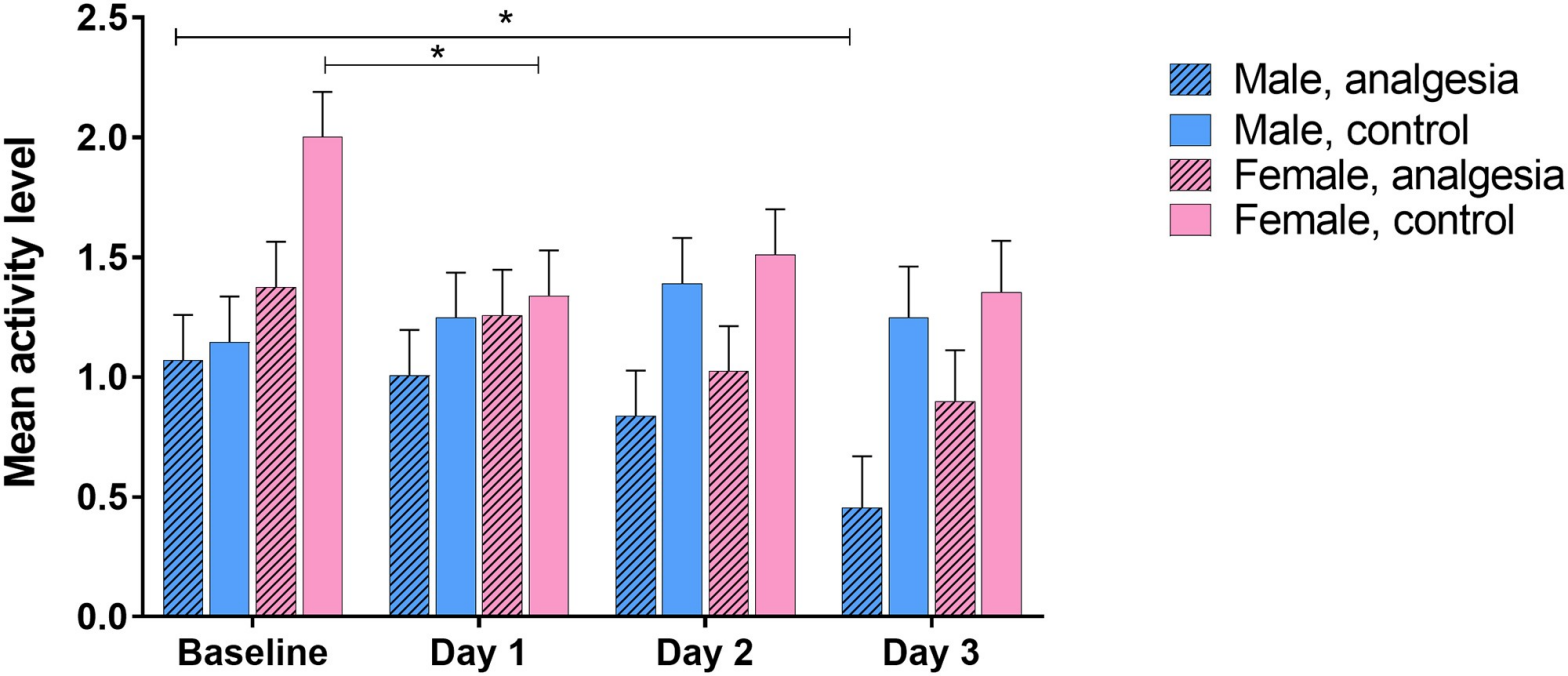
Activity level by day of experiment, sex, and analgesia treatment. Bars with asterisks indicate differences between categories (Tukey <0.05). Data were square root transformed for analysis; y-axis is back-transformed. Activity level is a linear measurement from 0 to 3; higher values indicate higher levels of activity. Data presented are LSM and SE.

Mean activity level analysis showed several significant factors. Light phase by day of experiment (GLM, F_(3, 184)_ = 8.41, P < 0.0001; Fig 5) indicated a decrease in activity during lights off for Days 1 and 3. During lights on, mice were more active on Day 1 than on Days 2 or 3. Additionally, sex by analgesia treatment by day of experiment (GLM, F_(3,184)_ = 3.64, P = 0.0139; Fig 6) demonstrated that female mice in the analgesia control group were less active on Day 1 than they were at baseline, and males in the analgesia treatment group were less active on Day 3 than at baseline. Finally, disruption treatment by analgesia treatment by light phase (GLM, F_(1, 184)_ = 5.85, P = 0.0166) showed only one difference—that mice in the unpredictable disruption plus analgesia control group were more active during lights off than lights on; there were no other differences between lights on/off or treatment groups.

### Sucrose consumption

Sucrose consumption, used as a measure of anhedonia or decreased affect, was affected by the main effect of sex (GLM, F_(1,24)_ = 5.49, P = 0.0277). Females were found to consume more sugared cereal than males. The day of the experiment was also significant (GLM, F_(2,50)_ = 10.78, P = 0.0001). Mice consumed more sucrose on Days 1 and 2 than at baseline. Bodyweight was included as a covariate, but was not significant.

### TINT

TINT success (a measure of general welfare) analysis had multiple significant effects. Mice at baseline were more likely to pass the TINT than on Days 1, 2, or 3 (GLIM, Χ^2^_(3)_ = 25.17, P < 0.0001). The interaction of sex by disruption treatment was significant (GLIM, Χ^2^_(1)_ = 6.82, P = 0.0090) but Bonferroni-corrected contrasts did not reveal any significant post-hoc comparisons. Sex by analgesia treatment interaction was also significant (GLIM, Χ^2^_(1)_ = 11.98, P = 0.0005). Males given analgesia were more likely to succeed than controls. Additionally, females in the control group were more likely to succeed than their male counterparts. Finally, disruption treatment by analgesia treatment (GLIM, Χ^2^_(1)_ = 7.84, P = 0.0051) was significant, but Bonferroni-corrected contrasts did not provide any significant comparisons.

### Food consumption

Total food consumption had two significant main effects, sex (GLM, F_(1, 20.91)_ = 4.99, P = 0.0366) and day of experiment (GLM, F_(3, 77.35)_ = 40.90, P < 0.0001). Female mice consumed more than males, and mice at baseline and Day 1 consumed more than those on Day 2 and Day 3. Bodyweight was a significant covariate (GLM, F_(1, 21.76)_ = 8.86, P = 0.007); as bodyweight increased, so did food consumption.

### Bodyweight

Bodyweight was affected by 3 main effects. Sex (GLM, F_(1, 27.71)_ = 53.26, P < 0.0001) showed that males were heavier than females. Day of experiment (GLM, F_(3,75)_ = 32.21, P < 0.0001) indicated that mice weighed more at baseline than Day 1, but less than on Days 2 and 3. Finally, disruption treatment (GLM, F_(1, 27.71)_ = 7.81, P = 0.0093) was significant, with mice in the unpredictable disruption group weighing more than those in the predictable group.

### Histopathology

There were no significant factors in either percent re-epithelialization or adrenal cortex:medulla ratio.

## Discussion

Few of our hypotheses (decreased proportion of time spent sleeping, shorter sleep bouts, decreased wound healing, decreased sucrose consumption, and increased adrenal cortex:medulla ratio) were supported by our results (Table 3). Proportion of time spent sleeping and sleep bout length were unaffected by predictability in disruption treatments, which was where we had expected to see the strongest results. This may be an example of anthropomorphism, where we as humans assumed that what we find unpleasant would also be aversive to the mice. While the investigator (ARJ) found stopping work constantly to conduct disruptions very frustrating and distracting, the mice did not seem to have been affected in the same way. However, it’s not clear whether the difference in perception by the mice was a matter of intensity of disruption, valence of disruption, or both. In fact, when compared to the percentage of time spent sleeping for C57BL/6 mice in our previous study with this sleep apparatus [14], it would appear that neither sleep disruption case had a strong effect.

**Table 3:** Measures, indication, hypotheses, and observations.

Measure	What it indicates	Hypothesized change	Observed change
Percentage of time sleeping	Level of sleep disruption	↓ percentage for unpredictable disruptions	No change, except for decrease on Day 1 post-op
Sleep bout length	Level of sleep fragmentation	↓ for unpredictable disruptions, ↓ for analgesia controls	↓ for female mice with analgesia
Activity level	Behavioral patterning changes	↓ post-op	↓ in unpredictable disruption x analgesia control group
TINT	Presence of pain or ↓ general welfare	↓ for controls and unpredictable disruptions post-op	↓ for analgesia controls
Sucrose preference	Anhedonia—negative effective state	↓ consumption for unpredictable disruptions and post-op	↑ consumption post-op
Re-epithelialization of wounds	Wound healing quality	↓ re-epithelialization for unpredictable disruptions and no analgesia	No difference
Adrenal cortex:medulla ratio	Chronic HPA axis activation	↑ ratio for unpredictable disruptions	No difference

Measures, what they indicate, our hypothesized changes, and observed changes.

Punch biopsies are used for wound healing studies[31, 34, 35, 70–74], and also for identification[61]. However, there is no consensus on analgesia protocols for mice who have had this procedure[31, 75, 76]. This seems to be a concern, since male mice who received analgesia spent more time sleeping than their female counterparts. This may be related to sex differences in pain perception. Females, in both humans and rodents, have been reported to perceive pain more intensely than males [77–80]. So while male mice might have experienced sufficient pain relief from the carprofen dosage, the females may not. This doesn’t explain why female mice were more likely to succeed in the TINT, which is an indicator of pain. It’s possible that female mice were, due to their lower bodyweight and increased heat loss to the environment, more motivated to nest build for thermoregulation in spite of their discomfort. In the future, either higher doses of analgesia or perhaps a combination of non-steroidal and opioid medication could achieve effective relief, particularly for female mice, without altering behavior.

Additionally, females who got analgesia had shorter sleep bout lengths than controls. This implies fragmentation of sleep for treated mice, which is unexpected, as decreased pain perception would be expected to improve sleep quality rather than decrease it. However, some early research into NSAIDs indicated that their administration may affect sleep quality, through moderation of prostaglandin production, inhibition of melatonin synthesis, and increased body temperature during sleep phases [81, 82]. This work has not been done in rodents, but perhaps a similar phenomenon occurred with our female mice.

Histopathology measures were unaffected by any of our treatments. The sleep disruptions, and subsequent stress that these treatments were meant to induce, may not have been sufficiently intense and/or prolonged enough to induce adrenal morphology changes, and were more acute than chronic. In studies where adrenal changes have been noted, durations of stressors have been at least 2 weeks[83–86], and when a stressor only lasted for one week, changes were not observed[87]. As far as the wound re-epithelialization, in rodents, wounds contract quickly due to their panniculus carnosus[35]. This is a layer of muscle that permits their skin to contract for healing. A wound splint process may have been helpful to prolong the healing process and more accurately assess re-epithelialization (more similarly to humans) [35]. However, it’s also possible that we didn’t sufficiently disrupt sleep in the mice, and therefore wound healing was not impaired.

Sucrose consumption results were also unexpected. We predicted that mice would have higher baseline consumption than any post-operative time point, regardless of treatments. Instead, we found exactly the opposite. Perhaps these mice required repeated exposures to overcome any food neophobia[88], needing time to learn that the cereal was highly palatable. Alternatively, this sucrose consumption pattern may be a reflection of how long mice actually need to acclimate to a new environment after transport. Baseline sucrose testing began for our mice approximately 3 days after arrival, with disruptions already occurring. These mice may not have been disturbed enough to change their sleeping patterns, but a decrease in general affect may have caused them to consume less sucrose.

Similarly our results for TINT success rate were lower than expected. The validation work on TINT demonstrated that mice at baseline were consistently successful after a few training tests [49, 64]; this was not the case for our mice. However, the mice in the referenced work had been present at the study facility for much longer than ours had (personal communication from BNG) and were almost certainly more acclimated to their environment.

One thing that was not surprising was the decrease in activity levels on Days 2 and 3. While perhaps counterintuitive, because presumably the mice were healing and should have been experiencing less pain, those days corresponded with the first restraint and injections the mice received. Mice responded negatively to these events, urinating, defecating, and vocalizing. This was the only time vocalizations were observed during the project. This suggests that the mice found the restraint extremely aversive, and their subsequent activity levels may be a reflection of that. We know that mice react differently to different types of handling[89], and that nest scores can be reduced after being handled by a novel individual[12]; this drop in activity may be a manifestation of their apparent aversion to unconditioned handling.

While our results didn’t support our hypotheses, they do raise some interesting questions regarding acclimation periods, sex differences in pain response, and just how disruptive human activity actually is to mice (particularly in regards to sleep). This project would suggest that direct interaction and restraint with the mice is more stressful than mere investigator presence or noise. However, this was only conducted with one strain of mice, over a relatively short time period. It is possible that mice in longer term projects may experience those events differently. At this time, we can’t make many recommendations, other than considering longer acclimation periods prior to commencing research, and investigating the longer term effects of carprofen use at higher doses for effective analgesia, particularly for female mice.

## Supporting information

S1 File Data set with SAS code for analyses performed. (DOCX)
