# Supplementary material for: Sleeping through anything: The effects of unpredictable disruptions on mouse sleep, healing, and affect
Source: PLoS One. 2019 Jan 31;14(1):e0210620. doi: 10.1371/journal.pone.0210620 (PMC6354982; doi:10.1371/journal.pone.0210620)
Supplement: S1 File — (DOCX) [file pone.0210620.s001.docx]

# Supplementary Data

The raw data or transformed data is provided for each analyzed measure (this can easily be viewed using the navigation pane in Word). Each data set is given as a SAS code for the data and the analysis itself. Data is presented in SAS format as this is a simple text format.

# Average percent asleep

DATA SleepPctByLightsAndPostOp; INPUT MouseName &$ Sex &$16. Cage SleepTx &$16. AnalgesiaTx &$16. Lights &$16. HoursPostOP &$16. AvgOfPercentSleep; Lines;

C1F1 F 1 Consolidated Analgesia Dark <24 0.191818181818182

C1F1 F 1 Consolidated Analgesia Dark 24 0.244375

C1F1 F 1 Consolidated Analgesia Dark -24 0.223636363636364

C1F1 F 1 Consolidated Analgesia Dark 48 0.31

C1F1 F 1 Consolidated Analgesia Light <24 0.518333333333333

C1F1 F 1 Consolidated Analgesia Light 24 0.604444444444444

C1F1 F 1 Consolidated Analgesia Light -24 0.559166666666667

C1F1 F 1 Consolidated Analgesia Light 48 0.543

C1F3 F 3 Consolidated Control Dark <24 0.358181818181818

C1F3 F 3 Consolidated Control Dark 24 0.31

C1F3 F 3 Consolidated Control Dark -24 0.379090909090909

C1F3 F 3 Consolidated Control Dark 48 0.429090909090909

C1F3 F 3 Consolidated Control Light <24 0.463333333333333

C1F3 F 3 Consolidated Control Light 24 0.563888888888889

C1F3 F 3 Consolidated Control Light -24 0.63

C1F3 F 3 Consolidated Control Light 48 0.679

C1F6 F 2 Consolidated Analgesia Dark <24 0.261818181818182

C1F6 F 2 Consolidated Analgesia Dark 24 0.294375

C1F6 F 2 Consolidated Analgesia Dark -24 0.380909090909091

C1F6 F 2 Consolidated Analgesia Dark 48 0.408181818181818

C1F6 F 2 Consolidated Analgesia Light <24 0.3675

C1F6 F 2 Consolidated Analgesia Light 24 0.556666666666667

C1F6 F 2 Consolidated Analgesia Light -24 0.469166666666667

C1F6 F 2 Consolidated Analgesia Light 48 0.524

C1F8 F 4 Consolidated Control Dark <24 0.298181818181818

C1F8 F 4 Consolidated Control Dark 24 0.266875

C1F8 F 4 Consolidated Control Dark -24 0.243636363636364

C1F8 F 4 Consolidated Control Dark 48 0.295454545454545

C1F8 F 4 Consolidated Control Light <24 0.389166666666667

C1F8 F 4 Consolidated Control Light 24 0.568888888888889

C1F8 F 4 Consolidated Control Light -24 0.545

C1F8 F 4 Consolidated Control Light 48 0.553

C1M2 M 2 Consolidated Analgesia Dark <24 0.399090909090909

C1M2 M 2 Consolidated Analgesia Dark 24 0.331875

C1M2 M 2 Consolidated Analgesia Dark -24 0.4

C1M2 M 2 Consolidated Analgesia Dark 48 0.466363636363636

C1M2 M 2 Consolidated Analgesia Light <24 0.431666666666667

C1M2 M 2 Consolidated Analgesia Light 24 0.701666666666667

C1M2 M 2 Consolidated Analgesia Light -24 0.6125

C1M2 M 2 Consolidated Analgesia Light 48 0.606

C1M4 M 4 Consolidated Control Dark <24 0.251818181818182

C1M4 M 4 Consolidated Control Dark 24 0.2525

C1M4 M 4 Consolidated Control Dark -24 0.346363636363636

C1M4 M 4 Consolidated Control Dark 48 0.202727272727273

C1M4 M 4 Consolidated Control Light <24 0.414166666666667

C1M4 M 4 Consolidated Control Light 24 0.599444444444444

C1M4 M 4 Consolidated Control Light -24 0.415

C1M4 M 4 Consolidated Control Light 48 0.669

C1M5 M 1 Consolidated Control Dark <24 0.330909090909091

C1M5 M 1 Consolidated Control Dark 24 0.385625

C1M5 M 1 Consolidated Control Dark -24 0.321818181818182

C1M5 M 1 Consolidated Control Dark 48 0.384545454545455

C1M5 M 1 Consolidated Control Light <24 0.426666666666667

C1M5 M 1 Consolidated Control Light 24 0.497222222222222

C1M5 M 1 Consolidated Control Light -24 0.493333333333333

C1M5 M 1 Consolidated Control Light 48 0.612

C1M7 M 3 Consolidated Analgesia Dark <24 0.396363636363636

C1M7 M 3 Consolidated Analgesia Dark 24 0.45375

C1M7 M 3 Consolidated Analgesia Dark -24 0.403636363636364

C1M7 M 3 Consolidated Analgesia Dark 48 0.562727272727273

C1M7 M 3 Consolidated Analgesia Light <24 0.564166666666667

C1M7 M 3 Consolidated Analgesia Light 24 0.612777777777778

C1M7 M 3 Consolidated Analgesia Light -24 0.6625

C1M7 M 3 Consolidated Analgesia Light 48 0.7

C2F2 F 2 Consolidated Control Dark <24 0.458181818181818

C2F2 F 2 Consolidated Control Dark 24 0.34125

C2F2 F 2 Consolidated Control Dark -24 0.29

C2F2 F 2 Consolidated Control Dark 48 0.353636363636364

C2F2 F 2 Consolidated Control Light <24 0.394166666666667

C2F2 F 2 Consolidated Control Light 24 0.492222222222222

C2F2 F 2 Consolidated Control Light -24 0.451666666666667

C2F2 F 2 Consolidated Control Light 48 0.512727272727273

C2F4 F 4 Consolidated Analgesia Dark <24 0.34

C2F4 F 4 Consolidated Analgesia Dark 24 0.295

C2F4 F 4 Consolidated Analgesia Dark -24 0.24

C2F4 F 4 Consolidated Analgesia Dark 48 0.280909090909091

C2F4 F 4 Consolidated Analgesia Light <24 0.3625

C2F4 F 4 Consolidated Analgesia Light 24 0.506111111111111

C2F4 F 4 Consolidated Analgesia Light -24 0.57

C2F4 F 4 Consolidated Analgesia Light 48 0.569090909090909

C2F5 F 1 Consolidated Analgesia Dark <24 0.315454545454545

C2F5 F 1 Consolidated Analgesia Dark 24 0.271875

C2F5 F 1 Consolidated Analgesia Dark -24 0.178181818181818

C2F5 F 1 Consolidated Analgesia Dark 48 0.137272727272727

C2F5 F 1 Consolidated Analgesia Light <24 0.313333333333333

C2F5 F 1 Consolidated Analgesia Light 24 0.465

C2F5 F 1 Consolidated Analgesia Light -24 0.471666666666667

C2F5 F 1 Consolidated Analgesia Light 48 0.457272727272727

C2F7 F 3 Consolidated Control Dark <24 0.364545454545455

C2F7 F 3 Consolidated Control Dark 24 0.374375

C2F7 F 3 Consolidated Control Dark -24 0.314545454545455

C2F7 F 3 Consolidated Control Dark 48 0.177272727272727

C2F7 F 3 Consolidated Control Light <24 0.4375

C2F7 F 3 Consolidated Control Light 24 0.461111111111111

C2F7 F 3 Consolidated Control Light -24 0.5275

C2F7 F 3 Consolidated Control Light 48 0.593636363636364

C2M1 M 1 Consolidated Analgesia Dark <24 0.348181818181818

C2M1 M 1 Consolidated Analgesia Dark 24 0.410625

C2M1 M 1 Consolidated Analgesia Dark -24 0.335454545454546

C2M1 M 1 Consolidated Analgesia Dark 48 0.448181818181818

C2M1 M 1 Consolidated Analgesia Light <24 0.445833333333333

C2M1 M 1 Consolidated Analgesia Light 24 0.566111111111111

C2M1 M 1 Consolidated Analgesia Light -24 0.6025

C2M1 M 1 Consolidated Analgesia Light 48 0.583636363636364

C2M3 M 3 Consolidated Control Dark <24 0.494545454545455

C2M3 M 3 Consolidated Control Dark 24 0.470625

C2M3 M 3 Consolidated Control Dark -24 0.28

C2M3 M 3 Consolidated Control Dark 48 0.400909090909091

C2M3 M 3 Consolidated Control Light <24 0.365833333333333

C2M3 M 3 Consolidated Control Light 24 0.536111111111111

C2M3 M 3 Consolidated Control Light -24 0.6025

C2M3 M 3 Consolidated Control Light 48 0.592727272727273

C2M6 M 2 Consolidated Analgesia Dark <24 0.421818181818182

C2M6 M 2 Consolidated Analgesia Dark 24 0.360625

C2M6 M 2 Consolidated Analgesia Dark -24 0.278181818181818

C2M6 M 2 Consolidated Analgesia Dark 48 0.230909090909091

C2M6 M 2 Consolidated Analgesia Light <24 0.494166666666667

C2M6 M 2 Consolidated Analgesia Light 24 0.651111111111111

C2M6 M 2 Consolidated Analgesia Light -24 0.548333333333333

C2M6 M 2 Consolidated Analgesia Light 48 0.606363636363636

C2M8 M 4 Consolidated Control Dark <24 0.307272727272727

C2M8 M 4 Consolidated Control Dark 24 0.431875

C2M8 M 4 Consolidated Control Dark -24 0.45

C2M8 M 4 Consolidated Control Dark 48 0.352727272727273

C2M8 M 4 Consolidated Control Light <24 0.381666666666667

C2M8 M 4 Consolidated Control Light 24 0.582777777777778

C2M8 M 4 Consolidated Control Light -24 0.496666666666667

C2M8 M 4 Consolidated Control Light 48 0.534545454545455

F1F2 F 2 Fragmented Analgesia Dark <24 0.458181818181818

F1F2 F 2 Fragmented Analgesia Dark 24 0.406875

F1F2 F 2 Fragmented Analgesia Dark -24 0.355454545454545

F1F2 F 2 Fragmented Analgesia Dark 48 0.433636363636364

F1F2 F 2 Fragmented Analgesia Light <24 0.460833333333333

F1F2 F 2 Fragmented Analgesia Light 24 0.565555555555556

F1F2 F 2 Fragmented Analgesia Light -24 0.526666666666667

F1F2 F 2 Fragmented Analgesia Light 48 0.544545454545455

F1F4 F 4 Fragmented Control Dark <24 0.257272727272727

F1F4 F 4 Fragmented Control Dark 24 0.2725

F1F4 F 4 Fragmented Control Dark -24 0.277272727272727

F1F4 F 4 Fragmented Control Dark 48 0.386363636363636

F1F4 F 4 Fragmented Control Light <24 0.461666666666667

F1F4 F 4 Fragmented Control Light 24 0.555555555555555

F1F4 F 4 Fragmented Control Light -24 0.565833333333333

F1F4 F 4 Fragmented Control Light 48 0.586363636363636

F1F5 F 1 Fragmented Control Dark <24 0.293636363636364

F1F5 F 1 Fragmented Control Dark 24 0.191875

F1F5 F 1 Fragmented Control Dark -24 0.185454545454545

F1F5 F 1 Fragmented Control Dark 48 0.272727272727273

F1F5 F 1 Fragmented Control Light <24 0.471666666666667

F1F5 F 1 Fragmented Control Light 24 0.607222222222222

F1F5 F 1 Fragmented Control Light -24 0.568333333333333

F1F5 F 1 Fragmented Control Light 48 0.602727272727273

F1F7 F 3 Fragmented Analgesia Dark <24 0.294545454545455

F1F7 F 3 Fragmented Analgesia Dark 24 0.346875

F1F7 F 3 Fragmented Analgesia Dark -24 0.255454545454545

F1F7 F 3 Fragmented Analgesia Dark 48 0.34

F1F7 F 3 Fragmented Analgesia Light <24 0.363333333333333

F1F7 F 3 Fragmented Analgesia Light 24 0.56

F1F7 F 3 Fragmented Analgesia Light -24 0.593333333333333

F1F7 F 3 Fragmented Analgesia Light 48 0.624545454545455

F1M1 M 1 Fragmented Control Dark <24 0.348181818181818

F1M1 M 1 Fragmented Control Dark 24 0.268125

F1M1 M 1 Fragmented Control Dark -24 0.262727272727273

F1M1 M 1 Fragmented Control Dark 48 0.317272727272727

F1M1 M 1 Fragmented Control Light <24 0.4175

F1M1 M 1 Fragmented Control Light 24 0.591111111111111

F1M1 M 1 Fragmented Control Light -24 0.580833333333333

F1M1 M 1 Fragmented Control Light 48 0.56

F1M3 M 3 Fragmented Analgesia Dark <24 0.542727272727273

F1M3 M 3 Fragmented Analgesia Dark 24 0.37625

F1M3 M 3 Fragmented Analgesia Dark -24 0.37

F1M3 M 3 Fragmented Analgesia Dark 48 0.388181818181818

F1M3 M 3 Fragmented Analgesia Light <24 0.485833333333333

F1M3 M 3 Fragmented Analgesia Light 24 0.605555555555556

F1M3 M 3 Fragmented Analgesia Light -24 0.624166666666667

F1M3 M 3 Fragmented Analgesia Light 48 0.663636363636364

F1M6 M 2 Fragmented Control Dark <24 0.279090909090909

F1M6 M 2 Fragmented Control Dark 24 0.4075

F1M6 M 2 Fragmented Control Dark -24 0.222727272727273

F1M6 M 2 Fragmented Control Dark 48 0.396363636363636

F1M6 M 2 Fragmented Control Light <24 0.44

F1M6 M 2 Fragmented Control Light 24 0.591111111111111

F1M6 M 2 Fragmented Control Light -24 0.631666666666667

F1M6 M 2 Fragmented Control Light 48 0.664545454545455

F1M8 M 4 Fragmented Analgesia Dark <24 0.276363636363636

F1M8 M 4 Fragmented Analgesia Dark 24 0.29625

F1M8 M 4 Fragmented Analgesia Dark -24 0.287272727272727

F1M8 M 4 Fragmented Analgesia Dark 48 0.320909090909091

F1M8 M 4 Fragmented Analgesia Light <24 0.3575

F1M8 M 4 Fragmented Analgesia Light 24 0.527222222222222

F1M8 M 4 Fragmented Analgesia Light -24 0.518333333333333

F1M8 M 4 Fragmented Analgesia Light 48 0.503636363636364

F2F1 F 1 Fragmented Control Dark <24 0.133636363636364

F2F1 F 1 Fragmented Control Dark 24 0.16125

F2F1 F 1 Fragmented Control Dark -24 0.257272727272727

F2F1 F 1 Fragmented Control Dark 48 0.298181818181818

F2F1 F 1 Fragmented Control Light <24 0.478333333333333

F2F1 F 1 Fragmented Control Light 24 0.484444444444444

F2F1 F 1 Fragmented Control Light -24 0.529166666666667

F2F1 F 1 Fragmented Control Light 48 0.49

F2F3 F 3 Fragmented Analgesia Dark <24 0.361818181818182

F2F3 F 3 Fragmented Analgesia Dark 24 0.37875

F2F3 F 3 Fragmented Analgesia Dark -24 0.345454545454545

F2F3 F 3 Fragmented Analgesia Dark 48 0.373636363636364

F2F3 F 3 Fragmented Analgesia Light <24 0.47

F2F3 F 3 Fragmented Analgesia Light 24 0.482222222222222

F2F3 F 3 Fragmented Analgesia Light -24 0.5425

F2F3 F 3 Fragmented Analgesia Light 48 0.558181818181818

F2F6 F 2 Fragmented Control Dark <24 0.325454545454546

F2F6 F 2 Fragmented Control Dark 24 0.368125

F2F6 F 2 Fragmented Control Dark -24 0.284545454545455

F2F6 F 2 Fragmented Control Dark 48 0.400909090909091

F2F6 F 2 Fragmented Control Light <24 0.539166666666667

F2F6 F 2 Fragmented Control Light 24 0.526666666666667

F2F6 F 2 Fragmented Control Light -24 0.560833333333333

F2F6 F 2 Fragmented Control Light 48 0.725454545454545

F2F8 F 4 Fragmented Analgesia Dark <24 0.313636363636364

F2F8 F 4 Fragmented Analgesia Dark 24 0.2225

F2F8 F 4 Fragmented Analgesia Dark -24 0.185454545454545

F2F8 F 4 Fragmented Analgesia Dark 48 0.218181818181818

F2F8 F 4 Fragmented Analgesia Light <24 0.385833333333333

F2F8 F 4 Fragmented Analgesia Light 24 0.499444444444444

F2F8 F 4 Fragmented Analgesia Light -24 0.4325

F2F8 F 4 Fragmented Analgesia Light 48 0.490909090909091

F2M2 M 2 Fragmented Control Dark <24 0.309090909090909

F2M2 M 2 Fragmented Control Dark 24 0.3325

F2M2 M 2 Fragmented Control Dark -24 0.257272727272727

F2M2 M 2 Fragmented Control Dark 48 0.318181818181818

F2M2 M 2 Fragmented Control Light <24 0.341666666666667

F2M2 M 2 Fragmented Control Light 24 0.568333333333333

F2M2 M 2 Fragmented Control Light -24 0.5225

F2M2 M 2 Fragmented Control Light 48 0.529090909090909

F2M4 M 4 Fragmented Analgesia Dark <24 0.474545454545455

F2M4 M 4 Fragmented Analgesia Dark 24 0.395625

F2M4 M 4 Fragmented Analgesia Dark -24 0.376363636363636

F2M4 M 4 Fragmented Analgesia Dark 48 0.510909090909091

F2M4 M 4 Fragmented Analgesia Light <24 0.523333333333333

F2M4 M 4 Fragmented Analgesia Light 24 0.643888888888889

F2M4 M 4 Fragmented Analgesia Light -24 0.515

F2M4 M 4 Fragmented Analgesia Light 48 0.661818181818182

F2M5 M 1 Fragmented Analgesia Dark <24 0.365454545454545

F2M5 M 1 Fragmented Analgesia Dark 24 0.431875

F2M5 M 1 Fragmented Analgesia Dark -24 0.398181818181818

F2M5 M 1 Fragmented Analgesia Dark 48 0.460909090909091

F2M5 M 1 Fragmented Analgesia Light <24 0.39

F2M5 M 1 Fragmented Analgesia Light 24 0.627222222222222

F2M5 M 1 Fragmented Analgesia Light -24 0.590833333333333

F2M5 M 1 Fragmented Analgesia Light 48 0.609090909090909

F2M7 M 3 Fragmented Control Dark <24 0.339090909090909

F2M7 M 3 Fragmented Control Dark 24 0.380625

F2M7 M 3 Fragmented Control Dark -24 0.276363636363636

F2M7 M 3 Fragmented Control Dark 48 0.409090909090909

F2M7 M 3 Fragmented Control Light <24 0.398333333333333

F2M7 M 3 Fragmented Control Light 24 0.526111111111111

F2M7 M 3 Fragmented Control Light -24 0.525833333333333

F2M7 M 3 Fragmented Control Light 48 0.574545454545455

;

RUN;

PROC MIXED ASYCOV NOBOUND DATA=SleepPctByLightsAndPostOp ALPHA=0.05;

CLASS MouseName Sex Cage SleepTx AnalgesiaTx Lights HoursPostOP;

MODEL AvgOfPercentSleep = Sex SleepTx AnalgesiaTx Lights HoursPostOP Sex*SleepTx Sex*AnalgesiaTx Sex*Lights Sex*HoursPostOP SleepTx*AnalgesiaTx SleepTx*Lights SleepTx*HoursPostOP AnalgesiaTx*Lights AnalgesiaTx*HoursPostOP Lights*HoursPostOP Sex*SleepTx*AnalgesiaTx Sex*SleepTx*Lights Sex*SleepTx*HoursPostOP Sex*AnalgesiaTx*Lights Sex*AnalgesiaTx*HoursPostOP Sex*Lights*HoursPostOP SleepTx*AnalgesiaTx*Lights SleepTx*AnalgesiaTx*HoursPostOP SleepTx*Lights*HoursPostOP AnalgesiaTx*Lights*HoursPostOP Cage/ SOLUTION DDFM=KENWARDROGER;

RANDOM MouseName(Sex SleepTx AnalgesiaTx ) / SOLUTION ;

RUN;

# Sleep bout length

DATA BoutLengthbyLightsandPostOp; INPUT MouseName &$ Sex &$16. SleepTx &$16. AnalgesiaTx &$16. Lights &$16. HoursPostOP &$16. SqRtBoutLength; Lines;

C1F1 F Consolidated Analgesia Dark <24 12.2944555131017

C1F1 F Consolidated Analgesia Dark 24 12.1346236410154

C1F1 F Consolidated Analgesia Dark -24 16.4017148493686

C1F1 F Consolidated Analgesia Dark 48 16.1756270076591

C1F1 F Consolidated Analgesia Light <24 18.9512752429311

C1F1 F Consolidated Analgesia Light 24 19.3436251342227

C1F1 F Consolidated Analgesia Light -24 26.6310386996494

C1F1 F Consolidated Analgesia Light 48 18.8781884724144

C1F3 F Consolidated Control Dark <24 15.4986802957249

C1F3 F Consolidated Control Dark 24 17.2876203736031

C1F3 F Consolidated Control Dark -24 19.35164721671

C1F3 F Consolidated Control Dark 48 19.5022609411879

C1F3 F Consolidated Control Light <24 16.8930952364174

C1F3 F Consolidated Control Light 24 27.6394193137265

C1F3 F Consolidated Control Light -24 24.4013888493622

C1F3 F Consolidated Control Light 48 33.2411341563431

C1F6 F Consolidated Analgesia Dark <24 11.9862800355619

C1F6 F Consolidated Analgesia Dark 24 13.6090544725328

C1F6 F Consolidated Analgesia Dark -24 14.3784126383965

C1F6 F Consolidated Analgesia Dark 48 21.5824423590524

C1F6 F Consolidated Analgesia Light <24 13.306608633808

C1F6 F Consolidated Analgesia Light 24 15.2057554892876

C1F6 F Consolidated Analgesia Light -24 20.8443837359931

C1F6 F Consolidated Analgesia Light 48 19.4418877684241

C1F8 F Consolidated Control Dark <24 16.9729731685942

C1F8 F Consolidated Control Dark 24 13.4688867056973

C1F8 F Consolidated Control Dark -24 19.4661886356832

C1F8 F Consolidated Control Dark 48 15.4574783313332

C1F8 F Consolidated Control Light <24 18.2603851730096

C1F8 F Consolidated Control Light 24 23.2941695137074

C1F8 F Consolidated Control Light -24 30.3092486801563

C1F8 F Consolidated Control Light 48 26.6849020983777

C1M2 M Consolidated Analgesia Dark <24 16.939196074087

C1M2 M Consolidated Analgesia Dark 24 15.3226807528756

C1M2 M Consolidated Analgesia Dark -24 17.9478759188936

C1M2 M Consolidated Analgesia Dark 48 18.2627788386404

C1M2 M Consolidated Analgesia Light <24 15.4052750705724

C1M2 M Consolidated Analgesia Light 24 23.0966808582244

C1M2 M Consolidated Analgesia Light -24 28.3522779802024

C1M2 M Consolidated Analgesia Light 48 25.3450784177126

C1M4 M Consolidated Control Dark <24 12.0020831525198

C1M4 M Consolidated Control Dark 24 14.161246735048

C1M4 M Consolidated Control Dark -24 14.3645396724016

C1M4 M Consolidated Control Dark 48 11.7438958999597

C1M4 M Consolidated Control Light <24 14.988745778083

C1M4 M Consolidated Control Light 24 19.60652527434

C1M4 M Consolidated Control Light -24 24.866733690705

C1M4 M Consolidated Control Light 48 24.642585091666

C1M5 M Consolidated Control Dark <24 14.2359659761018

C1M5 M Consolidated Control Dark 24 13.6148648709616

C1M5 M Consolidated Control Dark -24 15.851892631481

C1M5 M Consolidated Control Dark 48 18.5928872030531

C1M5 M Consolidated Control Light <24 16.7205960818786

C1M5 M Consolidated Control Light 24 19.1739406487034

C1M5 M Consolidated Control Light -24 19.5513142496582

C1M5 M Consolidated Control Light 48 26.2337187604045

C1M7 M Consolidated Analgesia Dark <24 16.5162729672064

C1M7 M Consolidated Analgesia Dark 24 14.911283098257

C1M7 M Consolidated Analgesia Dark -24 18.1212064995684

C1M7 M Consolidated Analgesia Dark 48 17.869425386295

C1M7 M Consolidated Analgesia Light <24 17.9234250819052

C1M7 M Consolidated Analgesia Light 24 29.7324177736468

C1M7 M Consolidated Analgesia Light -24 26.9297749299503

C1M7 M Consolidated Analgesia Light 48 31.369045251649

C2F2 F Consolidated Control Dark <24 24.3562946875975

C2F2 F Consolidated Control Dark 24 14.7674703933403

C2F2 F Consolidated Control Dark -24 20.6251666659933

C2F2 F Consolidated Control Dark 48 12.875805358748

C2F2 F Consolidated Control Light <24 15.242101779829

C2F2 F Consolidated Control Light 24 14.6662594640442

C2F2 F Consolidated Control Light -24 23.4464519940888

C2F2 F Consolidated Control Light 48 17.6543582051675

C2F4 F Consolidated Analgesia Dark <24 13.7372420151142

C2F4 F Consolidated Analgesia Dark 24 13.7107787723913

C2F4 F Consolidated Analgesia Dark -24 15.1088343362418

C2F4 F Consolidated Analgesia Dark 48 14.1760361173355

C2F4 F Consolidated Analgesia Light <24 18.0610307937651

C2F4 F Consolidated Analgesia Light 24 18.1506886921681

C2F4 F Consolidated Analgesia Light -24 19.1135786520706

C2F4 F Consolidated Analgesia Light 48 20.9175872065938

C2F5 F Consolidated Analgesia Dark <24 12.5227792442413

C2F5 F Consolidated Analgesia Dark 24 9.73456820726108

C2F5 F Consolidated Analgesia Dark -24 13.15596347669

C2F5 F Consolidated Analgesia Dark 48 9.43682342547726

C2F5 F Consolidated Analgesia Light <24 12.6822710899902

C2F5 F Consolidated Analgesia Light 24 15.2697686077207

C2F5 F Consolidated Analgesia Light -24 22.006488942027

C2F5 F Consolidated Analgesia Light 48 15.8899625264161

C2F7 F Consolidated Control Dark <24 18.0052265139371

C2F7 F Consolidated Control Dark 24 11.8362309725843

C2F7 F Consolidated Control Dark -24 13.4925210765075

C2F7 F Consolidated Control Dark 48 9.46347043780836

C2F7 F Consolidated Control Light <24 19.8892558935723

C2F7 F Consolidated Control Light 24 25.0701017149911

C2F7 F Consolidated Control Light -24 17.1332684823675

C2F7 F Consolidated Control Light 48 18.4104960182056

C2M1 M Consolidated Analgesia Dark <24 13.2445872306045

C2M1 M Consolidated Analgesia Dark 24 16.3512579443796

C2M1 M Consolidated Analgesia Dark -24 13.6682341580762

C2M1 M Consolidated Analgesia Dark 48 17.2237415426287

C2M1 M Consolidated Analgesia Light <24 15.6949567271359

C2M1 M Consolidated Analgesia Light 24 29.3451302036073

C2M1 M Consolidated Analgesia Light -24 23.9790996958231

C2M1 M Consolidated Analgesia Light 48 27.6742085375864

C2M3 M Consolidated Control Dark <24 19.2263550178584

C2M3 M Consolidated Control Dark 24 14.0361222306137

C2M3 M Consolidated Control Dark -24 20.4551491072541

C2M3 M Consolidated Control Dark 48 18.088519714298

C2M3 M Consolidated Control Light <24 17.8885438199983

C2M3 M Consolidated Control Light 24 21.2199591579877

C2M3 M Consolidated Control Light -24 20.1985285492671

C2M3 M Consolidated Control Light 48 26.7340436692449

C2M6 M Consolidated Analgesia Dark <24 16.9985560884126

C2M6 M Consolidated Analgesia Dark 24 10.87449058368

C2M6 M Consolidated Analgesia Dark -24 14.9657108083779

C2M6 M Consolidated Analgesia Dark 48 11.5605913658114

C2M6 M Consolidated Analgesia Light <24 22.9123147382945

C2M6 M Consolidated Analgesia Light 24 19.0778449167265

C2M6 M Consolidated Analgesia Light -24 28.991004351925

C2M6 M Consolidated Analgesia Light 48 24.2181562243927

C2M8 M Consolidated Control Dark <24 15.186118062829

C2M8 M Consolidated Control Dark 24 16.1610924473226

C2M8 M Consolidated Control Dark -24 17.5852566088755

C2M8 M Consolidated Control Dark 48 13.641047680372

C2M8 M Consolidated Control Light <24 14.3438430461761

C2M8 M Consolidated Control Light 24 17.4195292703333

C2M8 M Consolidated Control Light -24 22.1510220481534

C2M8 M Consolidated Control Light 48 18.3589710545504

F1F2 F Fragmented Analgesia Dark <24 18.7968807857242

F1F2 F Fragmented Analgesia Dark 24 14.772271321635

F1F2 F Fragmented Analgesia Dark -24 18.3497445486306

F1F2 F Fragmented Analgesia Dark 48 16.1875653736834

F1F2 F Fragmented Analgesia Light <24 16.2893370030827

F1F2 F Fragmented Analgesia Light 24 20.3557731368769

F1F2 F Fragmented Analgesia Light -24 24.0345006651318

F1F2 F Fragmented Analgesia Light 48 19.7580593452624

F1F4 F Fragmented Control Dark <24 14.0287367408992

F1F4 F Fragmented Control Dark 24 14.0880026328014

F1F4 F Fragmented Control Dark -24 16.0504478130674

F1F4 F Fragmented Control Dark 48 17.2436813196761

F1F4 F Fragmented Control Light <24 16.827111061221

F1F4 F Fragmented Control Light 24 23.5429925880292

F1F4 F Fragmented Control Light -24 25.6313826739366

F1F4 F Fragmented Control Light 48 22.346872874582

F1F5 F Fragmented Control Dark <24 26.3930087161526

F1F5 F Fragmented Control Dark 24 15.1054174508233

F1F5 F Fragmented Control Dark -24 14.5459401552461

F1F5 F Fragmented Control Dark 48 13.1773566117309

F1F5 F Fragmented Control Light <24 18.9270969776139

F1F5 F Fragmented Control Light 24 22.0608816686913

F1F5 F Fragmented Control Light -24 25.9399520259986

F1F5 F Fragmented Control Light 48 24.2256625765474

F1F7 F Fragmented Analgesia Dark <24 13.0469432156627

F1F7 F Fragmented Analgesia Dark 24 12.1841178290727

F1F7 F Fragmented Analgesia Dark -24 19.9193687148966

F1F7 F Fragmented Analgesia Dark 48 12.0541957991247

F1F7 F Fragmented Analgesia Light <24 16.9183086231061

F1F7 F Fragmented Analgesia Light 24 22.9252589952655

F1F7 F Fragmented Analgesia Light -24 27.1478871696819

F1F7 F Fragmented Analgesia Light 48 20.3741369030802

F1M1 M Fragmented Control Dark <24 16.5689468585061

F1M1 M Fragmented Control Dark 24 13.5186739532598

F1M1 M Fragmented Control Dark -24 16.9179638254726

F1M1 M Fragmented Control Dark 48 18.7494969629491

F1M1 M Fragmented Control Light <24 16.5702796194472

F1M1 M Fragmented Control Light 24 20.1186480659114

F1M1 M Fragmented Control Light -24 26.2016114856404

F1M1 M Fragmented Control Light 48 22.2342896527781

F1M3 M Fragmented Analgesia Dark <24 22.1531651750428

F1M3 M Fragmented Analgesia Dark 24 15.8298337440302

F1M3 M Fragmented Analgesia Dark -24 18.7295722054723

F1M3 M Fragmented Analgesia Dark 48 19.8189762142705

F1M3 M Fragmented Analgesia Light <24 19.1826093115613

F1M3 M Fragmented Analgesia Light 24 22.9432088717628

F1M3 M Fragmented Analgesia Light -24 29.0890300055995

F1M3 M Fragmented Analgesia Light 48 32.0452804637438

F1M6 M Fragmented Control Dark <24 21.0965098362911

F1M6 M Fragmented Control Dark 24 10.859181285061

F1M6 M Fragmented Control Dark -24 18.570423393127

F1M6 M Fragmented Control Dark 48 14.6111414525168

F1M6 M Fragmented Control Light <24 22.2866178083022

F1M6 M Fragmented Control Light 24 25.1045646579794

F1M6 M Fragmented Control Light -24 32.5296019034971

F1M6 M Fragmented Control Light 48 25.3307753892016

F1M8 M Fragmented Analgesia Dark <24 12.7568876226994

F1M8 M Fragmented Analgesia Dark 24 14.92228352011

F1M8 M Fragmented Analgesia Dark -24 14.058027066413

F1M8 M Fragmented Analgesia Dark 48 12.2539047580019

F1M8 M Fragmented Analgesia Light <24 13.5606354325058

F1M8 M Fragmented Analgesia Light 24 23.4447044482686

F1M8 M Fragmented Analgesia Light -24 20.7992120577252

F1M8 M Fragmented Analgesia Light 48 18.7063917127032

F2F1 F Fragmented Control Dark <24 10.3339686999192

F2F1 F Fragmented Control Dark 24 16.447934382607

F2F1 F Fragmented Control Dark -24 14.027383932865

F2F1 F Fragmented Control Dark 48 13.3364163102387

F2F1 F Fragmented Control Light <24 18.7069283065571

F2F1 F Fragmented Control Light 24 19.2007812341061

F2F1 F Fragmented Control Light -24 16.9695151505411

F2F1 F Fragmented Control Light 48 19.8715420090687

F2F3 F Fragmented Analgesia Dark <24 13.7464044885794

F2F3 F Fragmented Analgesia Dark 24 13.5207248326412

F2F3 F Fragmented Analgesia Dark -24 19.4427042872127

F2F3 F Fragmented Analgesia Dark 48 15.8909923026737

F2F3 F Fragmented Analgesia Light <24 14.1460536310779

F2F3 F Fragmented Analgesia Light 24 19.3222885118025

F2F3 F Fragmented Analgesia Light -24 17.2394734387232

F2F3 F Fragmented Analgesia Light 48 20.8255088336832

F2F6 F Fragmented Control Dark <24 19.6224129736659

F2F6 F Fragmented Control Dark 24 12.5643434875336

F2F6 F Fragmented Control Dark -24 17.3476763573684

F2F6 F Fragmented Control Dark 48 17.9953529354866

F2F6 F Fragmented Control Light <24 21.6625252452248

F2F6 F Fragmented Control Light 24 19.7043988320713

F2F6 F Fragmented Control Light -24 19.4423792554078

F2F6 F Fragmented Control Light 48 36.340485812533

F2F8 F Fragmented Analgesia Dark <24 11.6519994382548

F2F8 F Fragmented Analgesia Dark 24 10.9087537826696

F2F8 F Fragmented Analgesia Dark -24 15.2119607546167

F2F8 F Fragmented Analgesia Dark 48 10.7866499981142

F2F8 F Fragmented Analgesia Light <24 16.1799773382619

F2F8 F Fragmented Analgesia Light 24 14.9281892181648

F2F8 F Fragmented Analgesia Light -24 21.8430258384176

F2F8 F Fragmented Analgesia Light 48 17.0450739351446

F2M2 M Fragmented Control Dark <24 12.8317078860426

F2M2 M Fragmented Control Dark 24 11.4777254636179

F2M2 M Fragmented Control Dark -24 13.6290360994459

F2M2 M Fragmented Control Dark 48 16.9034423605479

F2M2 M Fragmented Control Light <24 12.2154615139994

F2M2 M Fragmented Control Light 24 15.9598715533678

F2M2 M Fragmented Control Light -24 19.3363647048767

F2M2 M Fragmented Control Light 48 21.0629791555965

F2M4 M Fragmented Analgesia Dark <24 16.2481047845867

F2M4 M Fragmented Analgesia Dark 24 16.9870271357024

F2M4 M Fragmented Analgesia Dark -24 15.1830703416667

F2M4 M Fragmented Analgesia Dark 48 17.2473713412166

F2M4 M Fragmented Analgesia Light <24 16.6852729475227

F2M4 M Fragmented Analgesia Light 24 19.2929823165489

F2M4 M Fragmented Analgesia Light -24 25.1837249031989

F2M4 M Fragmented Analgesia Light 48 25.9505824071971

F2M5 M Fragmented Analgesia Dark <24 16.6325802950933

F2M5 M Fragmented Analgesia Dark 24 17.2406496397323

F2M5 M Fragmented Analgesia Dark -24 19.2040197614979

F2M5 M Fragmented Analgesia Dark 48 15.9645345574609

F2M5 M Fragmented Analgesia Light <24 16.2208199546139

F2M5 M Fragmented Analgesia Light 24 29.0243719426737

F2M5 M Fragmented Analgesia Light -24 21.9321174941632

F2M5 M Fragmented Analgesia Light 48 22.0389943674223

F2M7 M Fragmented Control Dark <24 14.013564856952

F2M7 M Fragmented Control Dark 24 13.0214857412313

F2M7 M Fragmented Control Dark -24 17.5443901290413

F2M7 M Fragmented Control Dark 48 15.5536900035738

F2M7 M Fragmented Control Light <24 14.3170062047436

F2M7 M Fragmented Control Light 24 18.8937158865058

F2M7 M Fragmented Control Light -24 20.9692367264259

F2M7 M Fragmented Control Light 48 26.8793432008502

;

RUN;

PROC MIXED ASYCOV NOBOUND DATA=BoutLengthbyLightsandPostOp ALPHA=0.05;

CLASS MouseName Sex SleepTx AnalgesiaTx Lights HoursPostOP;

MODEL SqRtBoutLength = Sex SleepTx AnalgesiaTx Lights HoursPostOP Sex*SleepTx Sex*AnalgesiaTx Sex*Lights Sex*HoursPostOP SleepTx*AnalgesiaTx SleepTx*Lights SleepTx*HoursPostOP AnalgesiaTx*Lights AnalgesiaTx*HoursPostOP Lights*HoursPostOP Sex*SleepTx*AnalgesiaTx Sex*SleepTx*Lights Sex*SleepTx*HoursPostOP Sex*AnalgesiaTx*Lights Sex*AnalgesiaTx*HoursPostOP Sex*Lights*HoursPostOP SleepTx*AnalgesiaTx*Lights SleepTx*AnalgesiaTx*HoursPostOP SleepTx*Lights*HoursPostOP AnalgesiaTx*Lights*HoursPostOP/ SOLUTION DDFM=KENWARDROGER;

RANDOM MouseName(Sex SleepTx AnalgesiaTx ) / SOLUTION ;

RUN;

# Activity level

DATA ActivitybyLightsandPostOp; INPUT MouseName &$ Sex &$16. SleepTx &$16. AnalgesiaTx &$16. Lights &$16. HoursPostOP &$16. Sqrt_AvgOfHourlyActivity; Lines;

C1F1 F Consolidated Analgesia Dark <24 1.63262198486195

C1F1 F Consolidated Analgesia Dark 24 1.17512088353032

C1F1 F Consolidated Analgesia Dark -24 1.86681547025945

C1F1 F Consolidated Analgesia Dark 48 1.45133167689665

C1F1 F Consolidated Analgesia Light <24 1.31958277546303

C1F1 F Consolidated Analgesia Light 24 1.02209359878404

C1F1 F Consolidated Analgesia Light -24 1.06906781024685

C1F1 F Consolidated Analgesia Light 48 1.2035204769598

C1F3 F Consolidated Control Dark <24 1.02247471629109

C1F3 F Consolidated Control Dark 24 1.33926709943774

C1F3 F Consolidated Control Dark -24 1.42236598665744

C1F3 F Consolidated Control Dark 48 1.15836875899611

C1F3 F Consolidated Control Light <24 1.2724350313831

C1F3 F Consolidated Control Light 24 0.824936046320852

C1F3 F Consolidated Control Light -24 1.13818719263138

C1F3 F Consolidated Control Light 48 0.718438477640114

C1F6 F Consolidated Analgesia Dark <24 0.686890225449582

C1F6 F Consolidated Analgesia Dark 24 0.848528137423857

C1F6 F Consolidated Analgesia Dark -24 1.12943569980765

C1F6 F Consolidated Analgesia Dark 48 0.990408547473766

C1F6 F Consolidated Analgesia Light <24 1.02577180318422

C1F6 F Consolidated Analgesia Light 24 0.936767029486237

C1F6 F Consolidated Analgesia Light -24 0.810560637465542

C1F6 F Consolidated Analgesia Light 48 0.939230454230402

C1F8 F Consolidated Control Dark <24 1.62648533514894

C1F8 F Consolidated Control Dark 24 1.44064379547983

C1F8 F Consolidated Control Dark -24 2.00296654989542

C1F8 F Consolidated Control Dark 48 1.73152586413886

C1F8 F Consolidated Control Light <24 1.386437085557

C1F8 F Consolidated Control Light 24 1.27462752389681

C1F8 F Consolidated Control Light -24 1.46027160569298

C1F8 F Consolidated Control Light 48 1.30425339680483

C1M2 M Consolidated Analgesia Dark <24 1.66378538825829

C1M2 M Consolidated Analgesia Dark 24 1.1212817341207

C1M2 M Consolidated Analgesia Dark -24 0.942072184070839

C1M2 M Consolidated Analgesia Dark 48 0.893410218310614

C1M2 M Consolidated Analgesia Light <24 1.08268545395594

C1M2 M Consolidated Analgesia Light 24 1.0012978590796

C1M2 M Consolidated Analgesia Light -24 0.713843575002712

C1M2 M Consolidated Analgesia Light 48 0.831865373723422

C1M4 M Consolidated Control Dark <24 1.21655250605965

C1M4 M Consolidated Control Dark 24 1.12411581415634

C1M4 M Consolidated Control Dark -24 1.22856623753056

C1M4 M Consolidated Control Dark 48 1.4690751574313

C1M4 M Consolidated Control Light <24 1.02431478522023

C1M4 M Consolidated Control Light 24 1.28538235253477

C1M4 M Consolidated Control Light -24 0.873151282649891

C1M4 M Consolidated Control Light 48 0.696143221338385

C1M5 M Consolidated Control Dark <24 1.30905934305377

C1M5 M Consolidated Control Dark 24 1.34299529546323

C1M5 M Consolidated Control Dark -24 1.4596660576995

C1M5 M Consolidated Control Dark 48 1.15522528302727

C1M5 M Consolidated Control Light <24 1.2072830933681

C1M5 M Consolidated Control Light 24 1.21334570185409

C1M5 M Consolidated Control Light -24 1.20621609380627

C1M5 M Consolidated Control Light 48 0.991657509115225

C1M7 M Consolidated Analgesia Dark <24 1.16306804303414

C1M7 M Consolidated Analgesia Dark 24 1.18666836908288

C1M7 M Consolidated Analgesia Dark -24 1.19921849552114

C1M7 M Consolidated Analgesia Dark 48 0.917010954628728

C1M7 M Consolidated Analgesia Light <24 0.855084107657735

C1M7 M Consolidated Analgesia Light 24 0.915167999622185

C1M7 M Consolidated Analgesia Light -24 1.00817173954156

C1M7 M Consolidated Analgesia Light 48 0.75518719024338

C2F2 F Consolidated Control Dark <24 0.734228104263864

C2F2 F Consolidated Control Dark 24 1.21617881460372

C2F2 F Consolidated Control Dark -24 1.22244631783977

C2F2 F Consolidated Control Dark 48 1.09958669921516

C2F2 F Consolidated Control Light <24 1.42329520155491

C2F2 F Consolidated Control Light 24 1.39113493603475

C2F2 F Consolidated Control Light -24 1.21499731991267

C2F2 F Consolidated Control Light 48 1.1647007245545

C2F4 F Consolidated Analgesia Dark <24 1.03264884465322

C2F4 F Consolidated Analgesia Dark 24 0.996813103664052

C2F4 F Consolidated Analgesia Dark -24 1.04343183773546

C2F4 F Consolidated Analgesia Dark 48 0.853335700754292

C2F4 F Consolidated Analgesia Light <24 0.963899664956144

C2F4 F Consolidated Analgesia Light 24 0.625525420172555

C2F4 F Consolidated Analgesia Light -24 0.870332690180399

C2F4 F Consolidated Analgesia Light 48 0.645604798274883

C2F5 F Consolidated Analgesia Dark <24 0.83611656429646

C2F5 F Consolidated Analgesia Dark 24 1.09295263142803

C2F5 F Consolidated Analgesia Dark -24 1.04073291482494

C2F5 F Consolidated Analgesia Dark 48 0.999545351192688

C2F5 F Consolidated Analgesia Light <24 1.63620887639063

C2F5 F Consolidated Analgesia Light 24 1.10436198079044

C2F5 F Consolidated Analgesia Light -24 1.04185516501765

C2F5 F Consolidated Analgesia Light 48 1.04269948797447

C2F7 F Consolidated Control Dark <24 0.317661912902841

C2F7 F Consolidated Control Dark 24 1.04446593573419

C2F7 F Consolidated Control Dark -24 1.1008519428152

C2F7 F Consolidated Control Dark 48 1.37046773700873

C2F7 F Consolidated Control Light <24 0.751152959942758

C2F7 F Consolidated Control Light 24 0.785199631646922

C2F7 F Consolidated Control Light -24 1.19346962588042

C2F7 F Consolidated Control Light 48 0.855375161357088

C2M1 M Consolidated Analgesia Dark <24 1.07871977994119

C2M1 M Consolidated Analgesia Dark 24 1.04228769366411

C2M1 M Consolidated Analgesia Dark -24 0.923309265630966

C2M1 M Consolidated Analgesia Dark 48 0.705175671214435

C2M1 M Consolidated Analgesia Light <24 0.921537177345969

C2M1 M Consolidated Analgesia Light 24 0.906741135704944

C2M1 M Consolidated Analgesia Light -24 0.963231605612286

C2M1 M Consolidated Analgesia Light 48 0.675565606650377

C2M3 M Consolidated Control Dark <24 1.06173100513865

C2M3 M Consolidated Control Dark 24 2.02170045438802

C2M3 M Consolidated Control Dark -24 1.09344638643145

C2M3 M Consolidated Control Dark 48 1.58401101924542

C2M3 M Consolidated Control Light <24 1.39320696273943

C2M3 M Consolidated Control Light 24 1.41049182184342

C2M3 M Consolidated Control Light -24 1.13404163070325

C2M3 M Consolidated Control Light 48 1.27867118525444

C2M6 M Consolidated Analgesia Dark <24 0.394277244403663

C2M6 M Consolidated Analgesia Dark 24 0.635323825805677

C2M6 M Consolidated Analgesia Dark -24 1.05741429912783

C2M6 M Consolidated Analgesia Dark 48 0.41669696859512

C2M6 M Consolidated Analgesia Light <24 0.489505244727148

C2M6 M Consolidated Analgesia Light 24 0.668714802572432

C2M6 M Consolidated Analgesia Light -24 0.792930952730636

C2M6 M Consolidated Analgesia Light 48 0.338911656794381

C2M8 M Consolidated Control Dark <24 0.959166304662544

C2M8 M Consolidated Control Dark 24 0.632455532033675

C2M8 M Consolidated Control Dark -24 0.831790237980701

C2M8 M Consolidated Control Dark 48 0.869168882010019

C2M8 M Consolidated Control Light <24 0.981495457622369

C2M8 M Consolidated Control Light 24 0.87815365748475

C2M8 M Consolidated Control Light -24 0.791764331289633

C2M8 M Consolidated Control Light 48 0.732859695530686

F1F2 F Fragmented Analgesia Dark <24 0.972812230784357

F1F2 F Fragmented Analgesia Dark 24 1.23509292988606

F1F2 F Fragmented Analgesia Dark -24 1.46180539060437

F1F2 F Fragmented Analgesia Dark 48 0.9510759457296

F1F2 F Fragmented Analgesia Light <24 1.05957175674529

F1F2 F Fragmented Analgesia Light 24 1.10882242919558

F1F2 F Fragmented Analgesia Light -24 1.33768896411157

F1F2 F Fragmented Analgesia Light 48 0.923008366400026

F1F4 F Fragmented Control Dark <24 1.36181696807812

F1F4 F Fragmented Control Dark 24 1.624527903451

F1F4 F Fragmented Control Dark -24 1.86413518823072

F1F4 F Fragmented Control Dark 48 1.71835016010337

F1F4 F Fragmented Control Light <24 1.23454548887538

F1F4 F Fragmented Control Light 24 1.22923782931768

F1F4 F Fragmented Control Light -24 1.27480431848192

F1F4 F Fragmented Control Light 48 1.13063404040978

F1F5 F Fragmented Control Dark <24 1.17395988780785

F1F5 F Fragmented Control Dark 24 1.41260558736491

F1F5 F Fragmented Control Dark -24 1.81090447014745

F1F5 F Fragmented Control Dark 48 1.57451291169394

F1F5 F Fragmented Control Light <24 0.987550712751712

F1F5 F Fragmented Control Light 24 0.808369679330899

F1F5 F Fragmented Control Light -24 1.02997103655125

F1F5 F Fragmented Control Light 48 0.903234926989284

F1F7 F Fragmented Analgesia Dark <24 0.977938462462565

F1F7 F Fragmented Analgesia Dark 24 1.10247655097718

F1F7 F Fragmented Analgesia Dark -24 1.4609500333687

F1F7 F Fragmented Analgesia Dark 48 1.07449776851581

F1F7 F Fragmented Analgesia Light <24 1.20994808110694

F1F7 F Fragmented Analgesia Light 24 0.855225266470918

F1F7 F Fragmented Analgesia Light -24 1.10643647837202

F1F7 F Fragmented Analgesia Light 48 0.890848534326186

F1M1 M Fragmented Control Dark <24 0.945804131173815

F1M1 M Fragmented Control Dark 24 0.95679388870046

F1M1 M Fragmented Control Dark -24 1.4271912976192

F1M1 M Fragmented Control Dark 48 1.43019388386838

F1M1 M Fragmented Control Light <24 0.985341278896527

F1M1 M Fragmented Control Light 24 0.739715815147607

F1M1 M Fragmented Control Light -24 0.974377629415761

F1M1 M Fragmented Control Light 48 0.873927914647423

F1M3 M Fragmented Analgesia Dark <24 1.12653935088433

F1M3 M Fragmented Analgesia Dark 24 1.25625706691816

F1M3 M Fragmented Analgesia Dark -24 1.8214005600087

F1M3 M Fragmented Analgesia Dark 48 0.879566008686307

F1M3 M Fragmented Analgesia Light <24 1.3751922942461

F1M3 M Fragmented Analgesia Light 24 0.997432601668801

F1M3 M Fragmented Analgesia Light -24 1.4551560024948

F1M3 M Fragmented Analgesia Light 48 0.823356949728773

F1M6 M Fragmented Control Dark <24 1.10782341881399

F1M6 M Fragmented Control Dark 24 1.21916513908643

F1M6 M Fragmented Control Dark -24 1.10933538661669

F1M6 M Fragmented Control Dark 48 1.23103799513035

F1M6 M Fragmented Control Light <24 0.946044396421222

F1M6 M Fragmented Control Light 24 0.965029559797031

F1M6 M Fragmented Control Light -24 0.958070540025035

F1M6 M Fragmented Control Light 48 0.852121535410951

F1M8 M Fragmented Analgesia Dark <24 1.08040396485423

F1M8 M Fragmented Analgesia Dark 24 0.987190688036232

F1M8 M Fragmented Analgesia Dark -24 1.1

F1M8 M Fragmented Analgesia Dark 48 1.01891207578565

F1M8 M Fragmented Analgesia Light <24 0.978617549246025

F1M8 M Fragmented Analgesia Light 24 0.976978597251334

F1M8 M Fragmented Analgesia Light -24 0.794360373735591

F1M8 M Fragmented Analgesia Light 48 1.03016988890183

F2F1 F Fragmented Control Dark <24 1.57682073691451

F2F1 F Fragmented Control Dark 24 1.68144959105908

F2F1 F Fragmented Control Dark -24 1.75035710642144

F2F1 F Fragmented Control Dark 48 1.33688919782932

F2F1 F Fragmented Control Light <24 1.08012344973464

F2F1 F Fragmented Control Light 24 1.07315733546702

F2F1 F Fragmented Control Light -24 1.45290205098207

F2F1 F Fragmented Control Light 48 1.26309804317269

F2F3 F Fragmented Analgesia Dark <24 0.91502608010522

F2F3 F Fragmented Analgesia Dark 24 0.871779788708135

F2F3 F Fragmented Analgesia Dark -24 1.0074720839805

F2F3 F Fragmented Analgesia Dark 48 1.28275272186596

F2F3 F Fragmented Analgesia Light <24 1.04224855542335

F2F3 F Fragmented Analgesia Light 24 0.837961506979581

F2F3 F Fragmented Analgesia Light -24 0.863839387499179

F2F3 F Fragmented Analgesia Light 48 1.07399824125658

F2F6 F Fragmented Control Dark <24 0.869168882010018

F2F6 F Fragmented Control Dark 24 1.11599934832174

F2F6 F Fragmented Control Dark -24 1.12915897906362

F2F6 F Fragmented Control Dark 48 0.743456301827671

F2F6 F Fragmented Control Light <24 0.939926347360015

F2F6 F Fragmented Control Light 24 0.878810384729091

F2F6 F Fragmented Control Light -24 1.08360586961878

F2F6 F Fragmented Control Light 48 0.585709446360187

F2F8 F Fragmented Analgesia Dark <24 0.892392086676947

F2F8 F Fragmented Analgesia Dark 24 1.09045445071809

F2F8 F Fragmented Analgesia Dark -24 1.21166208160527

F2F8 F Fragmented Analgesia Dark 48 0.82296581172776

F2F8 F Fragmented Analgesia Light <24 1.27626257245371

F2F8 F Fragmented Analgesia Light 24 1.08030147739012

F2F8 F Fragmented Analgesia Light -24 0.96749698716227

F2F8 F Fragmented Analgesia Light 48 0.946117798632333

F2M2 M Fragmented Control Dark <24 0.631736423748788

F2M2 M Fragmented Control Dark 24 0.795441558335544

F2M2 M Fragmented Control Dark -24 0.976601249231227

F2M2 M Fragmented Control Dark 48 1.32012396112156

F2M2 M Fragmented Control Light <24 0.852447456836296

F2M2 M Fragmented Control Light 24 0.750726144202892

F2M2 M Fragmented Control Light -24 0.784460420455045

F2M2 M Fragmented Control Light 48 1.0080922576828

F2M4 M Fragmented Analgesia Dark <24 0.351619629196614

F2M4 M Fragmented Analgesia Dark 24 0.403394686042433

F2M4 M Fragmented Analgesia Dark -24 0.813172798364526

F2M4 M Fragmented Analgesia Dark 48 0.570486235990446

F2M4 M Fragmented Analgesia Light <24 0.496784532564041

F2M4 M Fragmented Analgesia Light 24 0.463265997689648

F2M4 M Fragmented Analgesia Light -24 0.785637887806018

F2M4 M Fragmented Analgesia Light 48 0.498051759916131

F2M5 M Fragmented Analgesia Dark <24 0.883176086632786

F2M5 M Fragmented Analgesia Dark 24 0.842614977317632

F2M5 M Fragmented Analgesia Dark -24 0.998123238883856

F2M5 M Fragmented Analgesia Dark 48 0.685565460040104

F2M5 M Fragmented Analgesia Light <24 1.14090607267499

F2M5 M Fragmented Analgesia Light 24 0.714142842854284

F2M5 M Fragmented Analgesia Light -24 0.832481357199086

F2M5 M Fragmented Analgesia Light 48 0.626054665699765

F2M7 M Fragmented Control Dark <24 1.4500783677882

F2M7 M Fragmented Control Dark 24 1.54596248337403

F2M7 M Fragmented Control Dark -24 1.1409973707244

F2M7 M Fragmented Control Dark 48 1.12573208503299

F2M7 M Fragmented Control Light <24 1.44413047212252

F2M7 M Fragmented Control Light 24 1.15208840472756

F2M7 M Fragmented Control Light -24 1.02021582821208

F2M7 M Fragmented Control Light 48 1.07050092116832

;

RUN;

PROC MIXED ASYCOV NOBOUND DATA=ActivitybyLightsandPostOp ALPHA=0.05;

CLASS MouseName Sex SleepTx AnalgesiaTx Lights HoursPostOP;

MODEL Sqrt_AvgOfHourlyActivity = Sex SleepTx AnalgesiaTx Lights HoursPostOP Sex*SleepTx Sex*AnalgesiaTx Sex*Lights Sex*HoursPostOP SleepTx*AnalgesiaTx SleepTx*Lights SleepTx*HoursPostOP AnalgesiaTx*Lights AnalgesiaTx*HoursPostOP Lights*HoursPostOP Sex*SleepTx*AnalgesiaTx Sex*SleepTx*Lights Sex*SleepTx*HoursPostOP Sex*AnalgesiaTx*Lights Sex*AnalgesiaTx*HoursPostOP Sex*Lights*HoursPostOP SleepTx*AnalgesiaTx*Lights SleepTx*AnalgesiaTx*HoursPostOP SleepTx*Lights*HoursPostOP AnalgesiaTx*Lights*HoursPostOP/ SOLUTION DDFM=KENWARDROGER;

RANDOM MouseName(Sex SleepTx AnalgesiaTx ) / SOLUTION ;

RUN;

# TINT

DATA TINTdataDays_1to3; INPUT MouseName &$ Sex &$16. SleepTx &$16. AnalgesiaTx &$16. GroupDay TINT_Success; Lines;

C1M2 M Consolidated Analgesia 0 1

C1F3 F Consolidated Control 0 1

C1M4 M Consolidated Control 0 0

C1M5 M Consolidated Control 0 0

C1F6 F Consolidated Analgesia 0 0

C1M7 M Consolidated Analgesia 0 1

C1F8 F Consolidated Control 0 1

C1F1 F Consolidated Analgesia 0 0

C1M2 M Consolidated Analgesia 1 1

C1F3 F Consolidated Control 1 1

C1M4 M Consolidated Control 1 0

C1M5 M Consolidated Control 1 0

C1F6 F Consolidated Analgesia 1 1

C1M7 M Consolidated Analgesia 1 0

C1F8 F Consolidated Control 1 1

C1F1 F Consolidated Analgesia 1 0

C1M2 M Consolidated Analgesia 2 0

C1F3 F Consolidated Control 2 1

C1M4 M Consolidated Control 2 0

C1M5 M Consolidated Control 2 0

C1F6 F Consolidated Analgesia 2 0

C1M7 M Consolidated Analgesia 2 0

C1F8 F Consolidated Control 2 0

C1F1 F Consolidated Analgesia 2 0

C1M2 M Consolidated Analgesia 3 0

C1F3 F Consolidated Control 3 1

C1M4 M Consolidated Control 3 0

C1M5 M Consolidated Control 3 0

C1F6 F Consolidated Analgesia 3 0

C1M7 M Consolidated Analgesia 3 0

C1F8 F Consolidated Control 3 1

C1F1 F Consolidated Analgesia 3 0

F1F4 F Fragmented Control 0 0

F1M1 M Fragmented Control 0 0

F1F2 F Fragmented Analgesia 0 1

F1M3 M Fragmented Analgesia 0 0

F1F5 F Fragmented Control 0 0

F1M6 M Fragmented Control 0 1

F1M8 M Fragmented Analgesia 0 1

F1F7 F Fragmented Analgesia 0 1

F1F4 F Fragmented Control 1 0

F1M1 M Fragmented Control 1 0

F1F2 F Fragmented Analgesia 1 1

F1M3 M Fragmented Analgesia 1 0

F1F5 F Fragmented Control 1 0

F1M6 M Fragmented Control 1 1

F1M8 M Fragmented Analgesia 1 0

F1F7 F Fragmented Analgesia 1 0

F1F4 F Fragmented Control 2 0

F1M1 M Fragmented Control 2 0

F1F2 F Fragmented Analgesia 2 0

F1M3 M Fragmented Analgesia 2 0

F1F5 F Fragmented Control 2 0

F1M6 M Fragmented Control 2 0

F1M8 M Fragmented Analgesia 2 0

F1F7 F Fragmented Analgesia 2 0

F1F4 F Fragmented Control 3 0

F1M1 M Fragmented Control 3 0

F1F2 F Fragmented Analgesia 3 0

F1M3 M Fragmented Analgesia 3 1

F1F5 F Fragmented Control 3 0

F1M6 M Fragmented Control 3 0

F1M8 M Fragmented Analgesia 3 0

F1F7 F Fragmented Analgesia 3 0

C2F7 F Consolidated Control 0 1

C2F2 F Consolidated Control 0 1

C2M3 M Consolidated Control 0 0

C2F4 F Consolidated Analgesia 0 1

C2M6 M Consolidated Analgesia 0 1

C2M1 M Consolidated Analgesia 0 1

C2M8 M Consolidated Control 0 0

C2F5 F Consolidated Analgesia 0 0

C2F7 F Consolidated Control 1 1

C2F2 F Consolidated Control 1 1

C2M3 M Consolidated Control 1 0

C2F4 F Consolidated Analgesia 1 0

C2M6 M Consolidated Analgesia 1 0

C2M1 M Consolidated Analgesia 1 0

C2M8 M Consolidated Control 1 0

C2F5 F Consolidated Analgesia 1 0

C2F7 F Consolidated Control 2 1

C2F2 F Consolidated Control 2 0

C2M3 M Consolidated Control 2 0

C2F4 F Consolidated Analgesia 2 0

C2M6 M Consolidated Analgesia 2 0

C2M1 M Consolidated Analgesia 2 0

C2M8 M Consolidated Control 2 0

C2F5 F Consolidated Analgesia 2 0

C2F7 F Consolidated Control 3 1

C2F2 F Consolidated Control 3 0

C2M3 M Consolidated Control 3 0

C2F4 F Consolidated Analgesia 3 0

C2M6 M Consolidated Analgesia 3 0

C2M1 M Consolidated Analgesia 3 0

C2M8 M Consolidated Control 3 0

C2F5 F Consolidated Analgesia 3 0

F2F8 F Fragmented Analgesia 0 1

F2F1 F Fragmented Control 0 0

F2M2 M Fragmented Control 0 0

F2F3 F Fragmented Analgesia 0 1

F2M4 M Fragmented Analgesia 0 0

F2M5 M Fragmented Analgesia 0 0

F2F6 F Fragmented Control 0 1

F2M7 M Fragmented Control 0 1

F2F8 F Fragmented Analgesia 1 0

F2F1 F Fragmented Control 1 0

F2M2 M Fragmented Control 1 0

F2F3 F Fragmented Analgesia 1 0

F2M4 M Fragmented Analgesia 1 1

F2M5 M Fragmented Analgesia 1 0

F2F6 F Fragmented Control 1 0

F2M7 M Fragmented Control 1 0

F2F8 F Fragmented Analgesia 2 0

F2F1 F Fragmented Control 2 0

F2M2 M Fragmented Control 2 0

F2F3 F Fragmented Analgesia 2 0

F2M4 M Fragmented Analgesia 2 1

F2M5 M Fragmented Analgesia 2 1

F2F6 F Fragmented Control 2 1

F2M7 M Fragmented Control 2 0

F2F8 F Fragmented Analgesia 3 0

F2F1 F Fragmented Control 3 0

F2M2 M Fragmented Control 3 0

F2F3 F Fragmented Analgesia 3 0

F2M4 M Fragmented Analgesia 3 0

F2M5 M Fragmented Analgesia 3 1

F2F6 F Fragmented Control 3 0

F2M7 M Fragmented Control 3 0

;

RUN;

PROC GENMOD DATA=TINTdataDays_1to3;

CLASS MouseName Sex SleepTx AnalgesiaTx GroupDay;

MODEL TINT_Success = MouseName(Sex SleepTx AnalgesiaTx ) Sex SleepTx AnalgesiaTx GroupDay Sex*SleepTx Sex*AnalgesiaTx Sex*GroupDay SleepTx*AnalgesiaTx SleepTx*GroupDay AnalgesiaTx*GroupDay Sex*SleepTx*AnalgesiaTx/ DIST=Binomial LINK=Logit ALPHA=0.05;

RUN;

# Sucrose consumption

DATA SucroseConsumption; INPUT MouseName &$ Sex &$16. SleepTx &$16. AnalgesiaTx &$16. GroupDay SucroseConsumed; Lines;

C1F1 F Consolidated Analgesia 1 2.2

C1F1 F Consolidated Analgesia 2 2.2

C1F3 F Consolidated Control -1 2.5

C1F3 F Consolidated Control 1 2.6

C1F3 F Consolidated Control 2 2.2

C1F6 F Consolidated Analgesia -1 2.2

C1F6 F Consolidated Analgesia 1 2.4

C1F6 F Consolidated Analgesia 2 2.7

C1F8 F Consolidated Control -1 2

C1F8 F Consolidated Control 1 2.2

C1F8 F Consolidated Control 2 2.2

C1M2 M Consolidated Analgesia -1 1.9

C1M2 M Consolidated Analgesia 1 2

C1M2 M Consolidated Analgesia 2 1.8

C1M4 M Consolidated Control -1 1.5

C1M4 M Consolidated Control 1 2.4

C1M4 M Consolidated Control 2 1.7

C1M5 M Consolidated Control -1 2.1

C1M5 M Consolidated Control 1 2.4

C1M5 M Consolidated Control 2 1.9

C1M7 M Consolidated Analgesia -1 2.3

C1M7 M Consolidated Analgesia 1 2.5

C1M7 M Consolidated Analgesia 2 2.1

C2F2 F Consolidated Control -1 1.5

C2F2 F Consolidated Control 1 2.2

C2F2 F Consolidated Control 2 2.3

C2F4 F Consolidated Analgesia -1 1.7

C2F4 F Consolidated Analgesia 1 2.8

C2F4 F Consolidated Analgesia 2 2.8

C2F5 F Consolidated Analgesia -1 2.6

C2F5 F Consolidated Analgesia 1 2.3

C2F5 F Consolidated Analgesia 2 2.4

C2F7 F Consolidated Control -1 2.2

C2F7 F Consolidated Control 1 2.6

C2F7 F Consolidated Control 2 1.9

C2M1 M Consolidated Analgesia -1 1.9

C2M1 M Consolidated Analgesia 1 2.6

C2M1 M Consolidated Analgesia 2 2.6

C2M3 M Consolidated Control -1 1.9

C2M3 M Consolidated Control 1 2.3

C2M3 M Consolidated Control 2 2.4

C2M6 M Consolidated Analgesia -1 2.1

C2M6 M Consolidated Analgesia 1 2.7

C2M6 M Consolidated Analgesia 2 2.5

C2M8 M Consolidated Control -1 1.2

C2M8 M Consolidated Control 1 1.6

C2M8 M Consolidated Control 2 2.2

F1F2 F Fragmented Analgesia -1 2.4

F1F2 F Fragmented Analgesia 1 2.9

F1F2 F Fragmented Analgesia 2 2.5

F1F4 F Fragmented Control -1 1.4

F1F4 F Fragmented Control 1 2

F1F4 F Fragmented Control 2 1.7

F1F5 F Fragmented Control -1 2

F1F5 F Fragmented Control 1 2.2

F1F5 F Fragmented Control 2 2

F1F7 F Fragmented Analgesia -1 2.7

F1F7 F Fragmented Analgesia 1 2.4

F1F7 F Fragmented Analgesia 2 2.2

F1M1 M Fragmented Control -1 1.8

F1M1 M Fragmented Control 1 2.4

F1M1 M Fragmented Control 2 2.1

F1M3 M Fragmented Analgesia -1 2.1

F1M3 M Fragmented Analgesia 1 3

F1M3 M Fragmented Analgesia 2 2.4

F1M6 M Fragmented Control -1 1.4

F1M6 M Fragmented Control 1 2

F1M6 M Fragmented Control 2 1.2

F1M8 M Fragmented Analgesia -1 1

F1M8 M Fragmented Analgesia 1 1.7

F1M8 M Fragmented Analgesia 2 2.2

F2F1 F Fragmented Control -1 2.3

F2F1 F Fragmented Control 1 2.8

F2F1 F Fragmented Control 2 2.5

F2F3 F Fragmented Analgesia -1 2

F2F3 F Fragmented Analgesia 1 2

F2F3 F Fragmented Analgesia 2 2.8

F2F6 F Fragmented Control -1 2.6

F2F6 F Fragmented Control 1 2.6

F2F6 F Fragmented Control 2 2.7

F2F8 F Fragmented Analgesia -1 2.4

F2F8 F Fragmented Analgesia 1 2.2

F2F8 F Fragmented Analgesia 2 2.1

F2M2 M Fragmented Control -1 1.2

F2M2 M Fragmented Control 1 1.9

F2M2 M Fragmented Control 2 2.1

F2M4 M Fragmented Analgesia -1 1.9

F2M4 M Fragmented Analgesia 1 2.9

F2M4 M Fragmented Analgesia 2 2.6

F2M5 M Fragmented Analgesia -1 1.6

F2M5 M Fragmented Analgesia 1 1.7

F2M5 M Fragmented Analgesia 2 2.3

F2M7 M Fragmented Control -1 2.5

F2M7 M Fragmented Control 1 2.1

F2M7 M Fragmented Control 2 2.3

C1F1 F Consolidated Analgesia -1 2.5

;

RUN;

PROC MIXED ASYCOV NOBOUND DATA=SucroseConsumption ALPHA=0.05;

CLASS MouseName Sex SleepTx AnalgesiaTx GroupDay;

MODEL SucroseConsumed = Sex SleepTx AnalgesiaTx GroupDay Sex*SleepTx Sex*AnalgesiaTx Sex*GroupDay SleepTx*AnalgesiaTx SleepTx*GroupDay AnalgesiaTx*GroupDay Sex*SleepTx*AnalgesiaTx Sex*SleepTx*GroupDay Sex*AnalgesiaTx*GroupDay SleepTx*AnalgesiaTx*GroupDay/ SOLUTION DDFM=KENWARDROGER;

RANDOM MouseName(Sex SleepTx AnalgesiaTx ) / SOLUTION ;

RUN;

# Total consumption

DATA TotalConsumptionAndBodyweightDat; INPUT MouseName &$ Sex &$16. Cage SleepTx &$16. AnalgesiaTx &$16. GroupDay Bodyweight TotalConsumption; Lines;

C1F1 F 1 Consolidated Analgesia 0 16.7 2.9

C1F3 F 3 Consolidated Control 0 15.9 1.3

C1F6 F 2 Consolidated Analgesia 0 17.5 2.1

C1F8 F 4 Consolidated Control 0 16.7 2

C1M2 M 2 Consolidated Analgesia 0 21.2 2.8

C1M4 M 4 Consolidated Control 0 20.8 3.7

C1M5 M 1 Consolidated Control 0 18.9 2.3

C1M7 M 3 Consolidated Analgesia 0 21.6 3.5

C2F2 F 2 Consolidated Control 0 16.4 2.2

C2F4 F 4 Consolidated Analgesia 0 18.6 3.5

C2F5 F 1 Consolidated Analgesia 0 19 3.2

C2F7 F 3 Consolidated Control 0 17.1 2.7

C2M1 M 1 Consolidated Analgesia 0 18.5 2.7

C2M3 M 3 Consolidated Control 0 19.5 1.7

C2M6 M 2 Consolidated Analgesia 0 18.8 3.1

C2M8 M 4 Consolidated Control 0 18.7 3.5

F1F2 F 2 Fragmented Analgesia 0 17.2 2.7

F1F4 F 4 Fragmented Control 0 17.7 2.9

F1F5 F 1 Fragmented Control 0 18.3 3.1

F1F7 F 3 Fragmented Analgesia 0 18.6 2

F1M1 M 1 Fragmented Control 0 20.9 2.5

F1M3 M 3 Fragmented Analgesia 0 22.8 3.3

F1M6 M 2 Fragmented Control 0 21.6 2.9

F1M8 M 4 Fragmented Analgesia 0 20 3.5

F2F1 F 1 Fragmented Control 0 19.2 2.5

F2F3 F 3 Fragmented Analgesia 0 17.9 2.1

F2F6 F 2 Fragmented Control 0 17.5 2.8

F2F8 F 4 Fragmented Analgesia 0 18.3 2

F2M2 M 2 Fragmented Control 0 20.5 3.1

F2M4 M 4 Fragmented Analgesia 0 20.9 3.6

F2M5 M 1 Fragmented Analgesia 0 19.3 3.1

F2M7 M 3 Fragmented Control 0 21.7 3.2

C1F1 F 1 Consolidated Analgesia 1 16.6 3.8

C1F3 F 3 Consolidated Control 1 15.4 3.8

C1F6 F 2 Consolidated Analgesia 1 17.3 3.4

C1F8 F 4 Consolidated Control 1 16.7 4.1

C1M2 M 2 Consolidated Analgesia 1 20.6 4

C1M4 M 4 Consolidated Control 1 20.8 4

C1M5 M 1 Consolidated Control 1 17.7 3.9

C1M7 M 3 Consolidated Analgesia 1 21.8 3.1

C2F2 F 2 Consolidated Control 1 16 3.5

C2F4 F 4 Consolidated Analgesia 1 18.5 4.4

C2F5 F 1 Consolidated Analgesia 1 18.9 5.3

C2F7 F 3 Consolidated Control 1 17.4 4.3

C2M1 M 1 Consolidated Analgesia 1 18.1 3.6

C2M3 M 3 Consolidated Control 1 17.2 5.1

C2M6 M 2 Consolidated Analgesia 1 18.8 4.2

C2M8 M 4 Consolidated Control 1 19.1 4.4

F1F2 F 2 Fragmented Analgesia 1 16.9 3.8

F1F4 F 4 Fragmented Control 1 17.6 3.8

F1F5 F 1 Fragmented Control 1 18.3 4.2

F1F7 F 3 Fragmented Analgesia 1 17.9 3.7

F1M1 M 1 Fragmented Control 1 20.5 3.4

F1M3 M 3 Fragmented Analgesia 1 22.9 4.7

F1M6 M 2 Fragmented Control 1 21 4.3

F1M8 M 4 Fragmented Analgesia 1 19.6 4.2

F2F1 F 1 Fragmented Control 1 18.6 4.1

F2F3 F 3 Fragmented Analgesia 1 17.4 3.6

F2F6 F 2 Fragmented Control 1 17.5 3.1

F2F8 F 4 Fragmented Analgesia 1 18.2 5.3

F2M2 M 2 Fragmented Control 1 21.1 3.8

F2M4 M 4 Fragmented Analgesia 1 20.7 3.8

F2M5 M 1 Fragmented Analgesia 1 19.4 2.9

F2M7 M 3 Fragmented Control 1 21.3 3.5

C1F1 F 1 Consolidated Analgesia 2 17.6 3.4

C1F3 F 3 Consolidated Control 2 16.2 2.8

C1F6 F 2 Consolidated Analgesia 2 17.7 3.9

C1F8 F 4 Consolidated Control 2 16.8 4.2

C1M2 M 2 Consolidated Analgesia 2 21 3.2

C1M4 M 4 Consolidated Control 2 21 5

C1M5 M 1 Consolidated Control 2 18.6 3.1

C1M7 M 3 Consolidated Analgesia 2 21.7 3.2

C2F2 F 2 Consolidated Control 2 16 3.7

C2F4 F 4 Consolidated Analgesia 2 19.1 4

C2F5 F 1 Consolidated Analgesia 2 19.1 4

C2F7 F 3 Consolidated Control 2 17.6 3.2

C2M1 M 1 Consolidated Analgesia 2 18.5 4.3

C2M3 M 3 Consolidated Control 2 19.7 3

C2M6 M 2 Consolidated Analgesia 2 19.1 3.2

C2M8 M 4 Consolidated Control 2 19.4 3.5

F1F2 F 2 Fragmented Analgesia 2 18 3.2

F1F4 F 4 Fragmented Control 2 18.1 3.1

F1F5 F 1 Fragmented Control 2 18.6 3.9

F1F7 F 3 Fragmented Analgesia 2 18.5 3.3

F1M1 M 1 Fragmented Control 2 21.4 2.9

F1M3 M 3 Fragmented Analgesia 2 23.6 3.7

F1M6 M 2 Fragmented Control 2 20.8 3.3

F1M8 M 4 Fragmented Analgesia 2 20.4 4.1

F2F1 F 1 Fragmented Control 2 19.3 3.9

F2F3 F 3 Fragmented Analgesia 2 18.4 3.6

F2F6 F 2 Fragmented Control 2 17.8 3.5

F2F8 F 4 Fragmented Analgesia 2 19.2 4.8

F2M2 M 2 Fragmented Control 2 21.3 3.2

F2M4 M 4 Fragmented Analgesia 2 21.1 3.3

F2M5 M 1 Fragmented Analgesia 2 19.9 3.6

F2M7 M 3 Fragmented Control 2 22.2 3.4

C1F1 F 1 Consolidated Analgesia -1 17.8 3.5

C1F3 F 3 Consolidated Control -1 16.5 4.8

C1F6 F 2 Consolidated Analgesia -1 18.1 5.7

C1F8 F 4 Consolidated Control -1 16.9 4.4

C1M2 M 2 Consolidated Analgesia -1 21.6 4.5

C1M4 M 4 Consolidated Control -1 21.2 3.9

C1M5 M 1 Consolidated Control -1 19.1 4.3

C1M7 M 3 Consolidated Analgesia -1 21.9 4

C2F2 F 2 Consolidated Control -1 16.1 3.8

C2F4 F 4 Consolidated Analgesia -1 19.2 4.2

C2F5 F 1 Consolidated Analgesia -1 18.9 5.4

C2F7 F 3 Consolidated Control -1 17.3 3.8

C2M1 M 1 Consolidated Analgesia -1 18.9 4

C2M3 M 3 Consolidated Control -1 20 3.6

C2M6 M 2 Consolidated Analgesia -1 19.6 3.4

C2M8 M 4 Consolidated Control -1 19.5 4.4

F1F2 F 2 Fragmented Analgesia -1 18.1 3.7

F1F4 F 4 Fragmented Control -1 18.1 4.5

F1F5 F 1 Fragmented Control -1 18.7 3.6

F1F7 F 3 Fragmented Analgesia -1 18.7 4.7

F1M1 M 1 Fragmented Control -1 20.8 4

F1M3 M 3 Fragmented Analgesia -1 23.9 5.1

F1M6 M 2 Fragmented Control -1 21 4.3

F1M8 M 4 Fragmented Analgesia -1 20.8 4.1

F2F1 F 1 Fragmented Control -1 19.1 4.4

F2F3 F 3 Fragmented Analgesia -1 18.7 3.6

F2F6 F 2 Fragmented Control -1 17.9 4.1

F2F8 F 4 Fragmented Analgesia -1 19.6 4.7

F2M2 M 2 Fragmented Control -1 21 5.9

F2M4 M 4 Fragmented Analgesia -1 21 4.3

F2M5 M 1 Fragmented Analgesia -1 20.4 4.4

F2M7 M 3 Fragmented Control -1 22.6 3.8

;

RUN;

PROC MIXED ASYCOV NOBOUND DATA=TotalConsumptionAndBodyweightDat ALPHA=0.05;

CLASS MouseName Sex Cage SleepTx AnalgesiaTx GroupDay;

MODEL TotalConsumption = Sex SleepTx AnalgesiaTx GroupDay Sex*SleepTx Sex*AnalgesiaTx Sex*GroupDay SleepTx*AnalgesiaTx SleepTx*GroupDay AnalgesiaTx*GroupDay Sex*SleepTx*AnalgesiaTx Sex*SleepTx*GroupDay Sex*AnalgesiaTx*GroupDay SleepTx*AnalgesiaTx*GroupDay Cage Bodyweight/ SOLUTION DDFM=KENWARDROGER;

RANDOM MouseName(Sex SleepTx AnalgesiaTx ) / SOLUTION ;

RUN;

# Bodyweight

DATA BodyweightByDay; INPUT MouseName &$ Sex &$16. SleepTx &$16. AnalgesiaTx &$16. GroupDay Weight; Lines;

C1F1 F Consolidated Analgesia 0 16.7

C1F1 F Consolidated Analgesia 1 16.6

C1F1 F Consolidated Analgesia 2 17.6

C1F1 F Consolidated Analgesia 3 17.8

C1F3 F Consolidated Control 0 15.9

C1F3 F Consolidated Control 1 15.4

C1F3 F Consolidated Control 2 16.2

C1F3 F Consolidated Control 3 16.5

C1F6 F Consolidated Analgesia 0 17.5

C1F6 F Consolidated Analgesia 1 17.3

C1F6 F Consolidated Analgesia 2 17.7

C1F6 F Consolidated Analgesia 3 18.1

C1F8 F Consolidated Control 0 16.7

C1F8 F Consolidated Control 1 16.7

C1F8 F Consolidated Control 2 16.8

C1F8 F Consolidated Control 3 16.9

C1M2 M Consolidated Analgesia 0 21.2

C1M2 M Consolidated Analgesia 1 20.6

C1M2 M Consolidated Analgesia 2 21

C1M2 M Consolidated Analgesia 3 21.6

C1M4 M Consolidated Control 0 20.8

C1M4 M Consolidated Control 1 20.8

C1M4 M Consolidated Control 2 21

C1M4 M Consolidated Control 3 21.2

C1M5 M Consolidated Control 0 18.9

C1M5 M Consolidated Control 1 17.7

C1M5 M Consolidated Control 2 18.6

C1M5 M Consolidated Control 3 19.1

C1M7 M Consolidated Analgesia 0 21.6

C1M7 M Consolidated Analgesia 1 21.8

C1M7 M Consolidated Analgesia 2 21.7

C1M7 M Consolidated Analgesia 3 21.9

C2F2 F Consolidated Control 0 16.4

C2F2 F Consolidated Control 1 16

C2F2 F Consolidated Control 2 16

C2F2 F Consolidated Control 3 16.1

C2F4 F Consolidated Analgesia 0 18.6

C2F4 F Consolidated Analgesia 1 18.5

C2F4 F Consolidated Analgesia 2 19.1

C2F4 F Consolidated Analgesia 3 19.2

C2F5 F Consolidated Analgesia 0 19

C2F5 F Consolidated Analgesia 1 18.9

C2F5 F Consolidated Analgesia 2 19.1

C2F5 F Consolidated Analgesia 3 18.9

C2F7 F Consolidated Control 0 17.1

C2F7 F Consolidated Control 1 17.4

C2F7 F Consolidated Control 2 17.6

C2F7 F Consolidated Control 3 17.3

C2M1 M Consolidated Analgesia 0 18.5

C2M1 M Consolidated Analgesia 1 18.1

C2M1 M Consolidated Analgesia 2 18.5

C2M1 M Consolidated Analgesia 3 18.9

C2M3 M Consolidated Control 0 19.5

C2M3 M Consolidated Control 1 17.2

C2M3 M Consolidated Control 2 19.7

C2M3 M Consolidated Control 3 20

C2M6 M Consolidated Analgesia 0 18.8

C2M6 M Consolidated Analgesia 1 18.8

C2M6 M Consolidated Analgesia 2 19.1

C2M6 M Consolidated Analgesia 3 19.6

C2M8 M Consolidated Control 0 18.7

C2M8 M Consolidated Control 1 19.1

C2M8 M Consolidated Control 2 19.4

C2M8 M Consolidated Control 3 19.5

F1F2 F Fragmented Analgesia 0 17.2

F1F2 F Fragmented Analgesia 1 16.9

F1F2 F Fragmented Analgesia 2 18

F1F2 F Fragmented Analgesia 3 18.1

F1F4 F Fragmented Control 0 17.7

F1F4 F Fragmented Control 1 17.6

F1F4 F Fragmented Control 2 18.1

F1F4 F Fragmented Control 3 18.1

F1F5 F Fragmented Control 0 18.3

F1F5 F Fragmented Control 1 18.3

F1F5 F Fragmented Control 2 18.6

F1F5 F Fragmented Control 3 18.7

F1F7 F Fragmented Analgesia 0 18.6

F1F7 F Fragmented Analgesia 1 17.9

F1F7 F Fragmented Analgesia 2 18.5

F1F7 F Fragmented Analgesia 3 18.7

F1M1 M Fragmented Control 0 20.9

F1M1 M Fragmented Control 1 20.5

F1M1 M Fragmented Control 2 21.4

F1M1 M Fragmented Control 3 20.8

F1M3 M Fragmented Analgesia 0 22.8

F1M3 M Fragmented Analgesia 1 22.9

F1M3 M Fragmented Analgesia 2 23.6

F1M3 M Fragmented Analgesia 3 23.9

F1M6 M Fragmented Control 0 21.6

F1M6 M Fragmented Control 1 21

F1M6 M Fragmented Control 2 20.8

F1M6 M Fragmented Control 3 21

F1M8 M Fragmented Analgesia 0 20

F1M8 M Fragmented Analgesia 1 19.6

F1M8 M Fragmented Analgesia 2 20.4

F1M8 M Fragmented Analgesia 3 20.8

F2F1 F Fragmented Control 0 19.2

F2F1 F Fragmented Control 1 18.6

F2F1 F Fragmented Control 2 19.3

F2F1 F Fragmented Control 3 19.1

F2F3 F Fragmented Analgesia 0 17.9

F2F3 F Fragmented Analgesia 1 17.4

F2F3 F Fragmented Analgesia 2 18.4

F2F3 F Fragmented Analgesia 3 18.7

F2F6 F Fragmented Control 0 17.5

F2F6 F Fragmented Control 1 17.5

F2F6 F Fragmented Control 2 17.8

F2F6 F Fragmented Control 3 17.9

F2F8 F Fragmented Analgesia 0 18.3

F2F8 F Fragmented Analgesia 1 18.2

F2F8 F Fragmented Analgesia 2 19.2

F2F8 F Fragmented Analgesia 3 19.6

F2M2 M Fragmented Control 0 20.5

F2M2 M Fragmented Control 1 21.1

F2M2 M Fragmented Control 2 21.3

F2M2 M Fragmented Control 3 21

F2M4 M Fragmented Analgesia 0 20.9

F2M4 M Fragmented Analgesia 1 20.7

F2M4 M Fragmented Analgesia 2 21.1

F2M4 M Fragmented Analgesia 3 21

F2M5 M Fragmented Analgesia 0 19.3

F2M5 M Fragmented Analgesia 1 19.4

F2M5 M Fragmented Analgesia 2 19.9

F2M5 M Fragmented Analgesia 3 20.4

F2M7 M Fragmented Control 0 21.7

F2M7 M Fragmented Control 1 21.3

F2M7 M Fragmented Control 2 22.2

F2M7 M Fragmented Control 3 22.6

;

RUN;

PROC MIXED ASYCOV NOBOUND DATA=BodyweightByDay ALPHA=0.05;

CLASS MouseName Sex SleepTx AnalgesiaTx GroupDay;

MODEL Weight = Sex SleepTx AnalgesiaTx GroupDay Sex*SleepTx Sex*AnalgesiaTx Sex*GroupDay SleepTx*AnalgesiaTx SleepTx*GroupDay AnalgesiaTx*GroupDay Sex*SleepTx*AnalgesiaTx Sex*SleepTx*GroupDay Sex*AnalgesiaTx*GroupDay SleepTx*AnalgesiaTx*GroupDay/ SOLUTION DDFM=KENWARDROGER;

RANDOM MouseName(Sex SleepTx AnalgesiaTx ) / SOLUTION ;

RUN;

# Percent re-epithelization

DATA EpithelializationData; INPUT MouseName &$ Sex &$16. SleepTx &$16. AnalgesiaTx &$16. Re_epithelialization_Values_; Lines;

C1F1 F Consolidated Analgesia 23.4621889345252

C1F1 F Consolidated Analgesia 15.4075583549463

C1M2 M Consolidated Analgesia 68.1882656350741

C1M2 M Consolidated Analgesia 29.8101874098991

C1F3 F Consolidated Control 38.1994301994302

C1M4 M Consolidated Control 16.1892130857648

C1M4 M Consolidated Control 37.9951338199513

C1M5 M Consolidated Control 15.3309186697399

C1M5 M Consolidated Control 16.5430656934307

C1F6 F Consolidated Analgesia 48.8411412469179

C1F6 F Consolidated Analgesia 23.9489126682775

C1M7 M Consolidated Analgesia .

C1F8 F Consolidated Control 23.6803057553957

C1F8 F Consolidated Control 31.6450822541127

F1M1 M Fragmented Control 31.6285777679166

F1M1 M Fragmented Control 100

F1F2 F Fragmented Analgesia 17.8752886836028

F1F2 F Fragmented Analgesia 38.8264794383149

F1F4 F Fragmented Control 81.7166748647319

F1F4 F Fragmented Control 29.3491255257915

F1F5 F Fragmented Control 18.3219512195122

F1M6 M Fragmented Control 79.8774259448417

F1M6 M Fragmented Control 30.2255022550226

F1F7 F Fragmented Analgesia 11.0860542335362

F1F7 F Fragmented Analgesia 100

F1M8 M Fragmented Analgesia 15.3274235058622

C2M1 M Consolidated Analgesia 35.6099151989563

C2M1 M Consolidated Analgesia 77.4012806830309

C2F2 F Consolidated Control 85.1168831168831

C2F2 F Consolidated Control 18.4310618066561

C2M3 M Consolidated Control 20.3216180371353

C2M3 M Consolidated Control 29.3864065449969

C2F4 F Consolidated Analgesia 26.6030825576072

C2F5 F Consolidated Analgesia 11.9313223297287

C2F5 F Consolidated Analgesia 18.3642224012893

C2M6 M Consolidated Analgesia 13.9185808505799

C2F7 F Consolidated Control 29.0544135429262

C2F7 F Consolidated Control 58.2922318125771

C2M8 M Consolidated Control 57.485837604532

F2F1 F Fragmented Control .

F2F1 F Fragmented Control 14.7930648769575

F2F1 F Fragmented Control 12.5671721202447

F2M2 M Fragmented Control 17.8072727272727

F2F3 F Fragmented Analgesia 78.3301946013811

F2M4 M Fragmented Analgesia 12.7552031714569

F2M5 M Fragmented Analgesia 23.1903785194854

F2F6 F Fragmented Control 34.2203742203742

F2F6 F Fragmented Control 38.5376242309513

F2M7 M Fragmented Control 25.3170163170163

F2M7 M Fragmented Control 19.5496582227583

F2F8 F Fragmented Analgesia 39.4699200673117

;

RUN;

PROC MIXED ASYCOV NOBOUND DATA=EpithelializationData ALPHA=0.05;

CLASS MouseName Sex SleepTx AnalgesiaTx;

MODEL Re_epithelialization_Values_ = Sex SleepTx Sex*SleepTx AnalgesiaTx Sex*AnalgesiaTx SleepTx*AnalgesiaTx Sex*SleepTx*AnalgesiaTx/ SOLUTION DDFM=KENWARDROGER;

RANDOM MouseName(Sex SleepTx AnalgesiaTx ) / SOLUTION ;

RUN;

# Adrenal cortex:medulla ratio

DATA AdrenalDataWithBodyweights; INPUT Sex &$16. SleepTx &$16. AnalgesiaTx &$16. Log_C_M_ratio; Lines;

M Consolidated Analgesia 0.0455794880733499

F Consolidated Control -0.202042607235081

M Consolidated Control -0.0836670899448709

M Consolidated Control 0.337133928007524

F Consolidated Analgesia -0.043692732476639

M Consolidated Analgesia -0.00484718354781989

F Consolidated Control 0.0413608813964558

F Consolidated Analgesia -0.0185988859997475

F Fragmented Control -0.197264000588429

M Fragmented Control -0.0529455011731004

F Fragmented Analgesia 0.0115616375569296

M Fragmented Analgesia 0.24748480643614

F Fragmented Control 0.208018334127607

M Fragmented Control 0.444525289701897

M Fragmented Analgesia -0.113848425222585

F Fragmented Analgesia 0.307365717386586

F Consolidated Control -0.06214523043855

F Consolidated Control -0.139487082012894

M Consolidated Control 0.0693099044802983

F Consolidated Analgesia 0.0731347186990351

M Consolidated Analgesia -0.236494446199126

M Consolidated Analgesia 0.0158370733790408

M Consolidated Control 0.0876777455256894

F Consolidated Analgesia 0.200557625509481

F Fragmented Analgesia 0.0484610869763682

F Fragmented Control -0.0264218619742039

M Fragmented Control 0.0287873196401171

F Fragmented Analgesia 0.119945618870475

M Fragmented Analgesia 0.00336454649956506

M Fragmented Analgesia -0.0170656376705828

F Fragmented Control 0.0272409676031761

M Fragmented Control -0.0954110992797627

;

RUN;

PROC GLM DATA=AdrenalDataWithBodyweights ALPHA=0.05;

CLASS Sex SleepTx AnalgesiaTx;

MODEL Log_C_M_ratio = Sex SleepTx Sex*SleepTx AnalgesiaTx Sex*AnalgesiaTx SleepTx*AnalgesiaTx Sex*SleepTx*AnalgesiaTx;

RUN;
